# The Impact of Different Drying Methods on the Metabolomic and Lipidomic Profiles of *Arthrospira platensis*

**DOI:** 10.3390/molecules29081747

**Published:** 2024-04-12

**Authors:** Marika Mróz, Karol Parchem, Joanna Jóźwik, M. Rosário Domingues, Barbara Kusznierewicz

**Affiliations:** 1 Department of Chemistry, Technology and Biotechnology of Food, Faculty of Chemistry, Gdańsk University of Technology, 11/12 Narutowicza St., 80-233 Gdańsk, Poland; marika.mroz@pg.edu.pl (M.M.); karol.parchem@pg.edu.pl (K.P.); joanna.jozwik@pg.edu.pl (J.J.); 2 Department of Analytical Chemistry, Faculty of Chemical Technology, University of Pardubice, Studentská 573, 532 10 Pardubice, Czech Republic; 3 Mass Spectrometry Centre, LAQV-REQUIMTE, Department of Chemistry, University of Aveiro, Santiago University Campus, 3810-193 Aveiro, Portugal; mrd@ua.pt; 4 Centre for Environmental and Marine Studies, CESAM, Department of Chemistry, University of Aveiro, Santiago University Campus, 3810-193 Aveiro, Portugal

**Keywords:** *Arthrospira platensis*, microalgae, metabolomics, lipidomics, HPLC-HRMS/MS, drying

## Abstract

Drying is an inseparable part of industrial microalgae production. In this work, the impacts of eight different drying methods on the metabolome and lipidome of *Arthrospira platensis* were investigated. The studied drying methods were freeze drying (FD), sun drying (SD), air drying at 40 and 75 °C (AD′ and AD″), infrared drying at 40 and 75 °C (IRD′ and IRD″), and vacuum drying at 40 and 75 °C (VD′ and VD″). Results gathered by reversed-phase liquid chromatography separation coupled with high-resolution tandem mass spectrometry with electrospray ionization (RP-LC-ESI-Orbitrap HRMS/MS) analysis allowed researchers to identify a total of 316 metabolites (including lipids) in aqueous and ethanolic extracts. The compounds identified in ethanolic extracts were mainly lipids, such as neutral and polar lipids, chlorophylls and carotenoids, while the compounds identified in the aqueous extracts were mainly amino acids and dipeptides. Among the identified compounds, products of enzymatic and chemical degradation, such as pyropheophytins, monoacylglycerols and lysophosphatidylcholines were also identified and their amounts depended on the drying method. The results showed that except for FD method, recognized as a control, the most protective method was AD′. Contrary to this, VD′ and VD″, under the conditions used, promoted the most intense degradation of valuable metabolites.

## 1. Introduction

*Arthrospira platensis*, known also as *Spirulina platensis*, is a genus of filamentous cyanobacteria (blue microalgae). Spirulina species are photosynthetic aquatic microorganisms; however, they can grow under either photoautotrophic, heterotrophic or mixotrophic conditions, which offers them high metabolic flexibility [1]. The natural habitats for *S. platensis* are alkaline water bodies, such as ponds and lakes, with a pH ranging from 9 to 11, that are rich in sodium carbonate and bicarbonate salts [2,3].

Spirulina is a natural source of many substances such as pigments, proteins and amino acids, and lipids, with various industrial applications. Pigments, such as phycobylinproteins, owing to their fluorescent properties, are used as markers in biomedicine, but they also serve as dyes in the food and cosmetics industries [4]. Another group of pigments, chlorophylls, are the substrate for the synthesis of chlorites, compounds used in photodynamic therapies against cancer [5]. Their content in spirulina can be up to 1% by its dry weight [6], which is over 10 times higher than that in any land plant studied so far [7]. Carotenoids, on the other hand, often serve as health-promoting substances. It is reported that carotenoid consumption prevents the development of cardiovascular diseases, neurodegenerative diseases, and cancer [8]. The content of carotenoids in *A. platensis* ranges from 0.15 to 0.2% of their dry biomass; one of the advantages of using them for carotenoid production is that they do not require as much agricultural intervention as land plants [9]. Spirulina is also of particular interest as a source of polyunsaturated fatty acids (PUFAs), the contents of which, in cyanobacteria, constitutes 20–60% of the total amount of fatty acids (FAs). PUFAs have beneficial health effects, such as those seen in the treatment of hypertension, premenstrual tension, and diabetes [10]. In addition, *A. platensis* biomass is rich in high-quality and highly bioavailable protein (over 50% of its dry biomass). It contains all the essential amino acids, making it an appropriate supplement to a vegetarian diet, as well as functional food components [11,12]. Moreover, as a source of branched-chained amino acids (BCAAs), spirulina is a promising alternative to whey proteins in the production of BCAA-containing peptides [13]. Thus, this microalgae is a valuable source of nutrients and phytochemicals for use in food, nutraceutical and pharmaceutical industries.

Nowadays, the cultivation of *A. platensis* and other microalgae, for industrial purposes, is mainly carried out in open ponds and less often in closed bioreactors [14]. The final stage of spirulina production, regardless of its intended use, is drying. The reduction of water content and water activity allows for the stabilization of spirulina biomass and, therefore, a much longer shelf life [15]. Its dried powder is also more commercially applicable as it could be easily processed into other forms such as pellets, or capsules. In the food, nutraceutical and dietary supplements industries, the drying of spirulina biomass, in addition to providing longer durability, is particularly important due to its increasing the concentrations of nutrients and health-beneficial compounds [16]. The drying of spirulina biomass is also used to induce cells to transform into an abiotic state for the needs of algological and biotechnological collections. According to Kharchuk et al. (2022) [17], dehydrating spirulina cells to a 7–11% residual humidity provided a highly viable cell biomass. In addition, they claim that the culture storage of *A. platensis* in this state provided long-term preservation with no negative effect on its biochemical composition. In their study, Kharchuk et al. (2022) [17] observed that the overall levels of proteins, lipids, carbohydrates, pigments and nucleic acid components did not change after the drying process, although the study did not show chemical modifications, which are likely to occur in dry biomass during drying and storage [15].

Despite the undeniable importance of drying in the industrial production of spirulina, this process strongly affects the quality of the final product; therefore, the technique and conditions should be carefully selected [18]. Freeze drying is considered to be the best method for preserving heat-sensitive products such as spirulina, but due to its high energy consumption it seems to be too expensive and less accessible for industrial purposes [19]. Besides freeze drying, the most common methods used for the drying of microalgae at an industrial level are solar drying and convective hot air drying, but they often cause the loss of unstable molecules such as phycocyanins, carotenoids and chlorophylls [20,21]. Another widely used technique for dehydrating microalgae is spray drying, but the cell disruption that accompanies this process and the high operating temperatures make the powder susceptible to oxidation and degradation [22]. However, a novel variant of this technique, namely electrostatic spray drying, may become a promising alternative as it operates at lower temperatures, with the advantage of preserving heat-sensitive compounds [23].

The main goal of the study was to determine the impact of different methods of microalgae drying on the metabolites and lipids in aqueous and ethanolic extracts of *A. platensis* using reversed-phase liquid chromatography separation coupled with high-resolution mass spectrometry with electrospray ionization (RP-LC-ESI-Orbitrap HRMS). The overall quality of the spirulina powders obtained with seven different drying methods was assessed based on the semi-quantitative analysis of the identified nutrients, non-nutritive bioactive compounds and their degradation products in comparison with freeze-dried spirulina.

## 2. Results and Discussion

### 2.1. The Comprehensive Metabolome-Wide Profiling of Freeze-Dried Spirulina 

**Figure 1:**
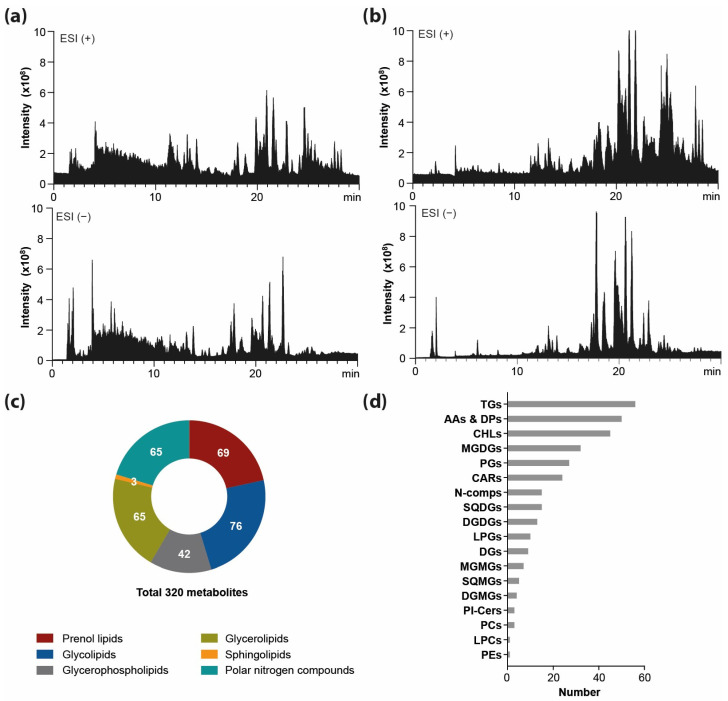
TIC of metabolite profiles of aqueous (**a**) and ethanolic (**b**) extracts from freeze-dried spirulina acquired in ESI(+) and ESI(−), set with a number of metabolites in groups of compounds (**c**) and classes of compounds (**d**) annotated in spirulina samples. (AAs & DPs, amino acids and dipeptides; CARs, carotenoids; CHLs, chlorophylls and derivatives; DGs, diacylglycerols; DGDGs, digalactosyl diacylglycerols; DGMGs, digalactosyl monoacylglycerols; LPCs, lysophosphatidylcholine; LPGs, lysophosphatidylglycerols; MGDGs, monogalactosyl diacylglycerols; MGMGs, monogalactosyl monoacylglycerols; N-comps, other polar nitrogen compounds; PCs, phosphatidylcholines; PEs, phosphatidylethanolamine; PGs, phosphatidylglycerols; Pl-Cers, ceramide phosphoinositols; SQDGs, sulfoquinovosyl diacylglycerols; SQMGs, sulfoquinovosyl monoacylglycerols; TGs, triacylglycerols).

High throughput HPLC-ESI-Orbitrap HRMS analysis of aqueous and ethanolic extracts obtained from freeze-dried spirulina biomass (Figure 1a,b) allowed for the identification and annotation of 316 metabolites in spirulina extracts (63 in aqueous and 253 in ethanolic extracts), confirmed by retention time, mass accuracy and MS/MS data matching. The metabolites and lipids spanned over six major groups of compounds, including prenol lipids, glycolipids, glycerophospholipids, glycerolipids, sphingolipids, and polar nitrogen compounds (Figure 1c), and within a given group of compounds, individual classes were distinguished. The identified metabolites included: 69 prenol lipids (45 chlorophylls and derivatives; 24 carotenoids), 76 glycolipids (32 monogalactosyl diacylglycerols, MGDGs; 7 monogalactosyl monoacylglycerols, MGMGs; 13 digalactosyl diacylglycerols, DGDGs; 4 digalactosyl monoacylglycerols, DGMGs; 15 sulfoquinovosyl diacylglycerols, SQDGs; 5 sulfoquinovosyl monoacylglycerols, SQMGs), 38 glycerophospholipids (3 phosphatidylcholines, PCs; 1 phosphatidylethanolamine, PEs; 27 phosphatidylglycerols, PGs; 1 lysophosphatidylcholine, LPC; 6 lysophosphatidylglycerols, LPGs), 65 glycerolipids (56 triacylglycerols, TGs; 9 diacylglycerols, DGs), 3 sphingolipids (3 ceramide phosphoinositols, PI-Cers) and 65 polar nitrogen compounds (13 amino acids, AAs; 37 dipeptides, DPs; 7 nucleobases, NBs; 6 other nitrogen compounds) (Figure 1d).

#### 2.1.1. The Profiling of Pigments

Among the wide range of compounds derived from spirulina, one of the most important groups is that of natural pigments, because in addition to their coloring potential, they also have health-promoting properties. There are three classes of pigments that are typical for spirulina: phycobiliproteins (14% of its dry weight), chlorophylls (1% of its dry weight), and carotenoids (0.37% of its dry weight) [6]. Table S1 lists the major pigments that were tentatively identified in aqueous and ethanolic crude extracts of *A. platensis* during untargeted analyses using HPLC/ESI-Orbitrap HRMS in positive ion mode, including phycobiliproteins, chlorophylls, carotenoids and their derivatives.

**Figure 2:**
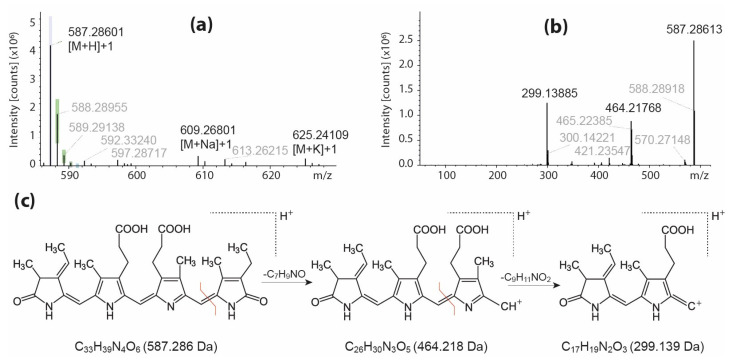
Characterization of the phycocyanobilin detected in spirulina extracts: (**a**) the LC-MS spectrum of phycocyanobilin (Compound **1**, RT = 11.8 min), (**b**) the MS/MS spectrum of the [M+H]^+^ ion of phycocyanobilin, and a (**c**) schematic representation of the formation of major fragment ions from phycocyanobilin.

Phycocyanin is a photosynthetic pigment protein, characteristic of cyanobacteria, used to harvest light and consisting of a protein residue and a linear tetrapyrrole chromophore, i.e., phycocyanobilin, connected by a thioether bond. The presented metabolomics analyses, which are limited to the profiling of small molecules, allowed for the detection of phycocyanobilin cleaved from phycocyanin in the tested spirulina extracts. Two compounds, (**1**) and (**17**), were assigned as phycocyanobilin isomers (Table S1). These compounds showed an [M+H]^+^ ion at *m*/*z* 587.3 (Figure 2a) and their identification was confirmed by the presence of the major fragment ions C_17_H_19_N_2_O_3_^+^ at *m*/*z* 299.1 and C_26_H_30_N_3_O_5_^+^ at *m*/*z* 464.2 observed in the MS/MS spectra (Figure 2b) and formed due to the successive loss of two pyrrole rings from the precursor ion (Figure 2c) that is consistent with other studies [24].

Carotenoids are also widely found in cyanobacteria. In the analyzed spirulina extracts, 24 compounds from this group were identified in both extracts (Table S1). They belong to different classes of carotenoids: hydroxyl, epoxy and ketone carotenoids or carotenes. All carotenoids were identified as protonated molecules [M+H]^+^, with the exception of *β*-carotene (**70**), which showed a molecular radical ion [M]^+•^ [25]. In all cases, the most intense fragment ions with low masses were observed in the MS/MS spectra, and formed a cluster of fragments from *m*/*z* at 93 to about 250 (e.g., *m*/*z* at 93, 95, 107, 109, 119, etc.) resulting from the fragmentation of the polyene skeleton, typical of carotenoids [26]. Other, less abundant fragmentation pathways were the loss of water [M+H−18]^+^ or the loss of toluene [M+H−92]^+^ molecules. Fragment ions typical of specific carotenoid pigments containing the same functional group, as reported by Rivera et al. (2014) [25], were also used to identify individual carotenoids. The fragment ion at *m*/*z* 203.1 was given by 3-hydroxyechinenone (**27**, **32**), canthaxanthin (**31**) and echinenone (**56**). This signal is a characteristic of carotenoids containing a keto group (as the only substituent on the *β*-ring) conjugated to the polyene chain. The fragment ion at *m*/*z* 135.1 was a characteristic of hydroxylated carotenoids, such as myxoxanthophyll (**15**), adonixanthin (**20**), antheraxanthin (**18**, **21**, **26**, **30**), diadinoxanthin (**23**) and zeaxanthin (**24**). The fragment ion at *m*/*z* 147.1 was observed in ketocarotenoids containing a hydroxyl group in carbon 3, (3′) and a keto group in carbon 4, (4′) in the β-ring such as adonirubin (**10**) and adonixanthin (**20**). Mass fragments at *m*/*z* 205.1 indicate the presence of an epoxy group as the only substituent on the β-ring such as in the case of *β*-cryptoxathin-5′,6′-epoxide (**41**). 

Chlorophylls and their dephytylated (chlorophyllide and pheophorbide) and phytylated (pheophytin) derivatives were also detected in the spirulina extracts. This included 45 compounds (Table S1), identified in LC-MS as [M+H]^+^ ions. The most abundant fragment ions in the MS/MS spectra of chlorophylls and their derivatives usually correspond to fragmentation with the loss of the phytyl chain (as the phytadiene, C_20_H_38_) or the loss of CH_3_COOC_20_H_39_ with the formation of the fragment ions [M+H−C_20_H_38_]^+^ = [M+H−278]^+^ and [M−CH_3_COOC_20_H_39_]^+^ = [M−338]^+^, respectively [27,28,29]. These fragment ions were observed in the cases of compounds **33**, **38**, **44**, **46**, **47**, **53**, **55**, **57**, **58**, **65**, and **66**. However, ions [M−C_20_H_38_]^+^ were present in the MS^2^ spectra of compounds **35**, **36**, **39**, **40**, **42**, **43**, **45**, **48**–**52**, **54**, **59**, **61**–**64**, **67**, and **68**. The loss of the CH_3_COOH group [M−60]^+^ was a characteristic of the fragmentation of dephytylated chlorophyll compounds such as in compounds **3**, **7**, **8**, **9**, **11**, and **12** [29,30].

#### 2.1.2. The Profiling of Lipids

The total lipid fraction of *Spirulina* spp. typically ranges to be between 5 and 14% of the dry matter and depends mainly on the growing conditions [31,32,33]. As reported by Couto et al. (2023), the lipidome of *A. platensis* includes mainly polar glycolipids such as sulfoquinovosyl diacylglycerols (SQDGs), monogalactosyl diacylglycerols (MGDGs), and digalactosyl diacylglycerols (DGDGs) [34]. Depending on the literature data source, glycolipids, which are major components of chloroplast and thylakoids membranes, represent between 30 and 50% of the total lipid fraction [31,35]. In addition to galactolipids, the prokaryotic cells of *Spirulina* spp. contain lysoglycolipids, namely sulfoquinovosyl monoacylglycerols (SQMGs), monogalactosyl monoacylglycerols (MGMGs) and digalactosyl monoacylglycerols (DGMGs), although in low abundances [36]. Using the RP-LC Orbitrap-HRMS/MS approach, 186 lipid species (excluding the prenol lipids described in Section 2.1.1) were identified in the ethanolic extracts of *A. platensis*, as shown in Tables S2–S7. 

According to the literature’s data, the most abundant class of glycolipids constitute SQDGs [34]. Together with their lyso forms, namely SQMGs, they were identified in negative ion mode as deprotonated ions. The MS/MS spectra of [M−H]^−^ ions corresponding to SQDGs and SQMGs revealed a characteristic fragmentation ion at *m*/*z* 225.0 consistent with the anion of the sulfoquinovosyl polar head group [37]. In total, 14 SQDG and 5 SQMG species were identified on a lipid species level in the ethanolic extracts of spirulina, as shown in Table S2. After SQDGs, MGDGs are the second most common class of glycolipid in *A. platensis* [34]. However, due to the lack of sulfonic acid residue in the structure, both MGDGs and MGMGs ionize preferentially in positive ion mode, as ammonium adducts, when compared to SQDG species. In the MS/MS spectra of [M+NH_4_^+^]^+^ ions corresponding to the MGDG class and its lyso form, the characteristic neutral loss of galactosyl moiety combined with the loss of NH_3_ (−197 Da) was observed [38]. Additionally, the characteristic ions [RCO+74]^+^, which are formed by the combined loss of FA and hexose moiety, was observed in the MS/MS spectra and allows for the identification of the FA composition of some MGDG species [39]. Among the identified lipids, 27 MGDG and 6 MGMG species characterized by various total carbon atom numbers of fatty acyl chain(s) and unsaturation degrees were found in the ethanolic spirulina extracts (Table S3). The DGDG class has been found to be the third most abundant class of glycolipid present in the prokaryotic cells of *A. platensis* [34,35]. Both DGDGs and DGMGs were identified in positive ion mode as ammonium adducts. The characteristic neutral loss observed in MS/MS spectra for DGDG and DGMG species is the loss of the digalactosyl polar head group combined with the loss of NH_3_ (−359 Da) [38]. Similarly to the MGDGs, [RCO+74]^+^ fragment ions enabled the identification of the FA present in the structure of DGDG species. In the studied ethanolic extracts, 14 DGDG and 3 DGMG species with FA(s) of various lengths and numbers of double bonds were identified, as shown in Table S3.

Phospholipids (PLs), another group of polar lipids identified in the prokaryotic cells of *A. platensis*, constitute around 20% of the total lipids [31]. Among them, phosphatidylglycerols (PGs) constitute by far the most abundant class of PLs [34]. PGs and lysophosphatidylglycerols (LPGs) were identified in negative ion mode as deprotonated ions. The MS/MS spectra of [M−H]^−^ ions, corresponding to PG and LPG species, showed a fragment ion at *m*/*z* 153.0, consistent with glycerol phosphate ion water loss, and fragment ion(s) [ROO]^−^**,** corresponding to the deprotonated FA(s) present in their structure [40]. Additionally, fragment ions at *m*/*z* 245.4, 227.0, and 171.0 corresponding to the glycerophosphoglycerol ion, the glycerophosphoglycerol ion with the loss of water, and the combined loss of the fatty acyl chain as ketene and glycerol from [M−H]^−^, respectively, were observed in the MS/MS spectra of LPG species [40]. In total, 16 PG and 5 LPG species were found, as shown in Table S4. For PG species, the lowest number of carbon atoms present in fatty acyl chains was 32, whereas the highest was 36. The total number of unsaturated bounds in the structure of FAs ranged being between 0 and 5. The profile of FAs present in LPG species included the following: palmitic acid (16:0), stearic acid (18:0), oleic acid (18:1), linoleic acid (18:2) and linolenic acid (18:3).

Other PL classes identified in the ethanolic extracts of *A. platensis* were phosphatidylcholines (PCs) and lysophosphatidylcholines (LPCs), as well as phosphatidylethanolamines (PEs). However, their species diversities were significantly lower compared to those of PG species, which can be related to the low abundance of these PL classes in the prokaryotic cells of *A. platensis*. PC, LPC and PE species were identified in positive ion mode as protonated ions [M+H]^+^. The MS/MS spectra corresponding to PC and LPE species revealed a typical fragmentation ion at *m*/*z* 184.1, consistent with the phosphocholine cation, whereas PE species showed a neutral loss of the phosphoethanolamine head group (−141 Da) [38]. Among the mentioned classes, the following PL species were found: PC (34:2), PC (36:2), and LPC (18:1) as well as PE (36:2) (Table S5).

The only one class of sphingolipids (SLs) identified in the extracts of *A. platensis* were ceramide phosphoinositol (PI-Cer) species, which were detected in negative ion mode as deprotonated ions. After fragmentation, ions at *m*/*z* 258.9 and 240.1, corresponding to the inositol phosphate ion and inositol phosphate ion with water loss, respectively, were observed in the MS/MS spectra. The following species were identified: PI-Cer (t18:0/16:0), PI-Cer (t18:0/18:0), and PI-Cer (t19:0/16:0) (Table S6). 

The polar lipidome of microalgae also includes betaine lipids (BLs), such as diacylglyceryl 3-O-4′-(N,N,N-trimethyl) homoserine (DGTS), diacylglycerylhydroxymethyl-N,N,N-trimethyl-β-alanine (DGTA) and their lyso forms [34]. However, BLs were not found in the studied ethanolic extracts of *A. platensis*. Firstly, prokaryotic cells of spirulina are characterized by relatively low levels of BLs compared to other microalgae species such as *Chlorococcum amblystomatis* or *Nannochloropsis oceanica* [34]. Secondly, in the cases of previous studies aimed at the characterization of the *A. platensis* lipidome, BLs lipids were detected in chloroform/methanol extracts [34]. This solvent mixture is known to be capable of extracting a wide spectrum of lipids with different polarities. Therefore, these two factors may have caused the absence of BLs in the studied ethanolic extracts.

Non-polar (neutral) lipids such as triacylglycerols (TGs) or diacylglycerols (DGs) are significant components of spirulina biomass and the typical amount of TGs corresponds to approximately 20% of the total lipids [31]. TG and DG species were identified in positive ion mode as ammonium adducts [41]. The structures of individual TG and DG species were confirmed based on the neutral loss of FAs present in their structures, combined with the loss of NH_3_, as observed in the MS/MS spectra of [M+NH_4_]^+^ ions. Among the identified neutral lipids, 47 TG and 5 DG species were found in the ethanolic extract of spirulina (Table S7). TG (34:0) species were characterized by a lower number of total carbon atoms in fatty acyl chains, while among the saturated TG species, the highest number of carbon atoms in chains was observed for TG (58:0). In turn, the most unsaturated identified TG species was TG (54:6). In this case, the following possible FAs were found: linoleic acid (18:2), docosahexaenoic acid (22:6, DHA), eicosapentaenoic acid (20:5, EPA), linolenic acid (18:3), and oleic acid (18:1). The total carbon atom chain length in DG species ranged from 32 to 34 atoms, while DG (34:4) was found to be the most unsaturated species. Interestingly, in the ethanolic extract of *A. platensis*, monoacylglycerol (MG) species were not detected. This can result from the specificity of spirulina lipase, which was found to release FA only from the *sn* − 3 position of the glycerol backbone, leading to the formation of 1,2-DGs, but not 2,3-DGs and MG species [42].

#### 2.1.3. The Profiling of Polar Nitro-Organic Compounds

Polar nitro-organic compounds are the most abundant compounds in spirulina. Among them, proteins may constitute up to 70% of the dry biomass, which makes spirulina one of the most nutritious microalgae. However, the nutritional quality of algal protein is determined not only by the content, but also by proportion and availability of amino acids. Amino acids profile of spirulina depend on many factors including cultivation conditions and harvest time [43,44]. Another compounds containing nitrogen are free nucleotides, which constitute up to 3% of spirulina dry mass [17]. In this study, MS analysis in positive ionization mode have been used to detect and identify low molecular weight polar nutrients such as free amino acids, dipeptides and nucleic acids components. The identification of these compounds was performed by comparing the observed exact masses with the calculated masses and was further confirmed by comparison with the spectra of metabolomic standards available in the Mass bank (https://massbank.eu/MassBank/ (accessed on 1 September 2023)) and Human Metabolome database (https://hmdb.ca/ (accessed on 12 September 2023)). In total, 13 amino acids, 37 dipeptides, 7 nucleobases and 6 other nitro-organic compounds have been identified in studied spirulina extracts (Table S8). 

Amino acids’ fragmentation starts with specific cleavage reactions at the C-terminal side of the molecule and the N-terminal side resulting in the formation of characteristic fragments. Major fragments usually result from the loss of H_2_O + CO (−46 Da) and NH_3_ (−17 Da) fragment loss, or sequential losses of NH_3_ (−17 Da), H_2_O (−18 Da), CO (−28 Da) or CH_2_CO (−42 Da) fragments [45]. Further fragmentation resulting from the presence of specific side-chains enables a more-confident identification of amino acids. Compound **255** and **258**, with [M+H]^+^ ions at *m*/*z* 147.1 and 175.1, shared a fragment ion at *m*/*z* 84.1 and were identified as lysine and N,N-dimethyl lysine. Compound **257** yielded the base peak [M+H]^+^ at *m*/*z* 156.1 and a fragment ion, characteristic for histidine, at *m*/*z* 110.1, arising from a loss of H_2_O and CO. Compounds **260** and **262** ([M+H]^+^ at *m*/*z* 147.1 and 148.1) shared characteristic fragments at *m*/*z* 84.0 and 56.0, and thus were identified as glutamine and glutamic acid. The fragmentation pattern (*m*/*z* at 70, 60, 71 and 72) of the compound that showed a precursor ion at *m*/*z* 175.1 suggested that compound **261** was arginine. Compound **279**, with an [M+H]^+^ at *m*/*z* 150.0, gave fragments similar to those in the spectrum obtained for the methionine standard by LC-ESI-QTOF MS2 analysis provided in massbank.eu by Research Unit Analytical BioGeoChemistry (BGC), Helmholtz Zentrum München. Compound **266** ([M+H]^+^ at *m*/*z* 166.0) was identified as methionine sulfoxide as it showed the same characteristic fragment ion at *m*/*z* 102.0 as methionine. Compound **291**, with an [M+H]^+^ at *m*/*z* 132.1 was designated as leucine or isoleucine due to the presence of characteristic fragments at *m*/*z* 86.1 and 69.1 formed by the loss of H_2_O + CO (−46 Da) and NH_3_ (−17 Da), respectively. The compound 304 ([M+H]^+^ at *m*/*z* 166.1) gave the fragment ions (*m*/*z* at 103, 120, 91, 95, 79 and 93) that were in line with those of phenylalanine. Compound **309** ([M+H]^+^ at *m*/*z* 205.1) produced the fragment ions (*m*/*z* at 118, 143, 132, 115, 144 and 142) that are typical for tryptophan. 

The majority of the polar nitro-organic compounds detected in spirulina extracts were dipeptides. Among them, eleven compounds with fragments which are characteristics for leucine (*m*/*z* at 86 and 69) were detected and identified. Compound **302** ([M+H]^+^ at *m*/*z* 260.2) was identified as glutaminyl-leucine and compound **305** ([M+H]^+^ at *m*/*z* 229.1) was identified as leucyl-proline. Other compounds with leucyl fragments, marked with the following numbers: **280**, **282**, **284**, **303**, **304**, **310**, **312**, **314** and **315**, can be found in Supplementary Table S8. The fragment ion at *m*/*z* 116.1 formed by the elimination of the guanidine group (CH_5_N_3_) was one of the ions that allowed for the identification of several dipeptides (**263**, **264**, **265**, **276**, **283** and **293**), as this particular cleavage starts one of the two major fragmentation patterns of arginine [45]. Dipeptides that contain histidine gave the common fragment ion of protonated histidine (*m*/*z* at 156.1) and a fragment ion at *m*/*z* 110.1 arising from a loss of H_2_O and CO. Hence, compounds **273**, **275** and **282** with an [M+H]^+^ at *m*/*z* 255.1, 253.1 and 269.2 were identified as valyl-histidine, histidyl-proline and histidyl-leucine, respectively. In addition to compound **273**, eight other compounds (**272**, **274**, **276**, **278**, **295**, **299**, **310** and **313** in Supplementary Table S8) gave characteristic fragments at *m*/*z* 72.1 and 55.0, and as a result of which these were designated as dipeptides containing valine. The dipeptides that contain proline gave a common fragment ion at *m*/*z* 116.1 (Pro) and a fragment that can be associated with the loss of CH_2_O_2_ from proline (*m*/*z* at 70.1), as was previously reported by Ma et al. (2013) [46]. Hence, compound **277** ([M+H]^+^ at *m*/*z* 173.1) was identified as prolyl-glycine and compound **286** with a precursor ion at *m*/*z* 187.1 was identified as alanyl-proline. The same fragments were also observed in compounds **288** and **293**, identified as glutaminyl-proline and hydroxyprolyl-arginine, respectively. All essential amino acids were detected in the tested aqueous spirulina extracts, in free form (His, Leu, Ile, Lys, Met, Phe, and Trp) or in the form of dipeptides (Val and Thr).

Another class of compounds identified in aqueous spirulina extracts were nucleobases, which are one of the building blocks of DNA. Compounds **271** and **287** shared a characteristic fragment at *m*/*z* 112.0 and, therefore, were identified as cytidine diphosphate and cytidine, respectively. The fragmentation pattern of compound **289** with a parent ion [M+H]^+^ at *m*/*z* 152.0, was in accordance with that of the guanine reported by Weimann et al. (2002) [47]. Based on the presence of a fragment corresponding to guanine in compound **297**, it was identified as its derivative, namely, guanosine monophosphate. Compound **294** ([M+H]^+^ at *m*/*z* 136.1) was identified as adenine as it produced characteristic fragment ions at *m*/*z* 119.0 and 109.0, formed by the loss of NH_3_ and HCN, respectively [48]. Additionally, fragments at *m*/*z* 136 and 119 were present in the MS/MS spectrum of compound **301**, which was identified as 2′-deoxyadenosine. Thymine, the last of the DNA nucleobases, was not observed in the studied extracts. However, there was a signal with a pseudomolecular ion at *m*/*z* 265.1, which could be associated with thymidine, which is a deoxynucleoside, but it was not confirmed with the MS/MS spectrum, hence, this compound was not included in the identification table. The detailed MS data and tentative identifications of the observed amino acids, dipeptides and other polar nitro-organic compounds are summarized in Supplementary Table S8.

### 2.2. The Modulation of Metabolomic Profiles of Spirulina after Drying

To show the impact of the drying methods on spirulina metabolome, a lipidome RP-LC-ESI-Orbitrap HRMS analysis of aqueous and ethanolic extracts prepared from differently dried spirulina (FD, freeze-dried; SD, sun-dried; AD′, air-dried at 40 °C; AD″, air-dried at 75 °C; IRD′, infrared-dried at 40 °C; IRD″, infrared-dried at 75 °C; VD′, vacuum-dried at 40 °C; VD″, vacuum-dried at 75 °C) was performed. The differentiation of dried spirulina samples was carried out based on the variability of the content of metabolites previously identified in the freeze-dried spirulina, which was treated as a control method in the case of the ethanol extract for the quantification of carotenoids, lipids and their degradation products, and in the case of the water extracts for quantification of polar nitro-organic compounds. 

A partial least squares discriminant analysis (PLS-DA) and a hierarchal cluster analysis (HCA) were performed on the normalized peak areas of all 320 identified metabolites. For the HCA, normalized peak areas were summed within the appropriate classes. 

**Figure 3:**
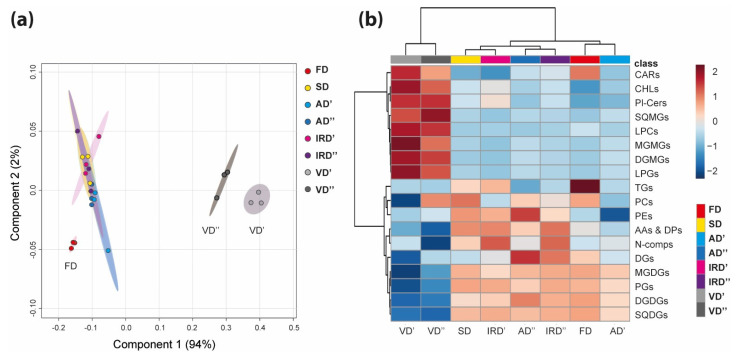
Global metabolic features of the studied dried spirulina powders. (**a**) The score plot represents the partial least squares discriminant analysis (PLS-DA) of LC-MS data. Ellipses represent clusters (95% confidence regions); A (**b**) heatmap visualization of 18 metabolites classes after the drying of the spirulina. The red-blue color scale indicates the summed up peaks intensity being higher or lower than the average. Each column indicates a different drying method (FD, freeze drying; SD, sun drying; AD′, air drying at 40 °C; AD″, air drying at 75 °C; IRD′, infrared drying at 40 °C; IRD″, infrared drying at 75 °C; VD′, vacuum drying at 40 °C; VD″, vacuum drying at 75 °C) and each row indicates a different metabolite class (AAs & DPs, amino acids and dipeptides; CARs, carotenoids; CHLs, chlorophylls and derivatives; DGs, diacylglycerols; DGDGs, digalactosyl diacylglycerols; DGMGs, digalactosyl monoacylglycerols; LPCs, lysophosphatidylcholine; LPGs, lysophosphatidylglycerols; MGDGs, monogalactosyl diacylglycerols; MGMGs, monogalactosyl monoacylglycerols; N-comps, other polar nitrogen compounds; PCs, phosphatidylcholines; PEs, phosphatidylethanolamine; PGs, phosphatidylglycerols; Pl-Cers, ceramide phosphoinositols; SQDGs, sulfoquinovosyl diacylglycerols; SQMGs, sulfoquinovosyl monoacylglycerols; TGs, triacylglycerols).

PLS-DA resulted in the spatial visualization of the metabolite profiles of the studied spirulina extracts (Figure 3a). The first two principal components (PC1 and PC2) in aqueous and ethanolic compounds explained 94% and 2% of the total variance. The PC1/PC2 score plots show that eight clusters, corresponding to the different drying methods studied, are formed and distributed over three regions. The clusters formed by FD, VD′ and VD″ were sufficiently separated, while the other clusters overlapped to some extent. Given the relatively mild conditions, freeze drying is expected to induce lesser alteration in the *A. platensis* metabolome compared to the other drying methods, while the reason of forming such distant clusters in the case of the vacuum drying methods is not clear.

As a result of the HCA of all extracts, the drying methods were divided in two main clusters (Figure 3b). The first cluster included six drying methods (IRD′, IRD″, AD′, AD″, FD and SD), while the second cluster included two drying methods (VD′ and VD″). Row clustering revealed two sets of substance classes, both strongly influenced by the VD′ and VD″ drying methods, but in the opposite manner. In the following sections, we will describe in detail the main differences in each class of metabolites depending on the drying method used.

#### 2.2.1. The Impacts of Drying Methods on Pigments

**Figure 4:**
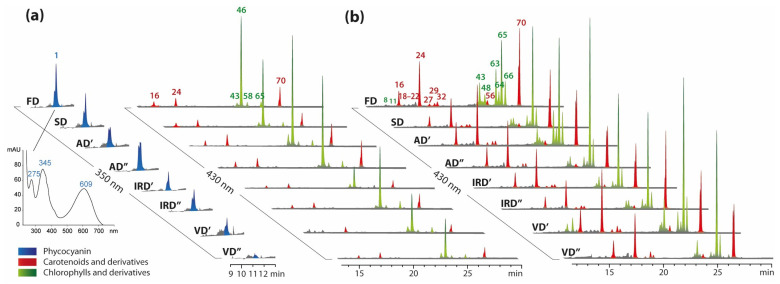
The HPLC-PAD chromatograms of pigments present in aqueous (**a**) and ethanolic (**b**) extracts prepared from spirulina dried using different methods (FD, freeze drying; SD, sun drying; AD′, air drying at 40 °C; AD″, air drying at 75 °C; IRD′, infrared drying at 40 °C; IRD″, infrared drying at 75 °C; VD′, vacuum drying at 40 °C; VD″, vacuum drying at 75 °C) and registered at 350 and 430 nm. The peak numbers correspond to the compound numbers in Table S1.

The color of spirulina extracts varied mainly depending on the solvent used for extraction. Aqueous extracts were dark blue, while ethanolic extracts were dark green. These visual differences indicated the presence of blue phycocyanin and green chlorophylls in the aqueous and alcoholic extracts, respectively. This was additionally confirmed by chromatograms recorded using the PAD detector, namely LC-PAD (Figure 4a,b).

Only the chromatograms of aqueous spirulina extracts indicated the presence of a compound with a retention time between 11 and 12 min and a characteristic absorption spectrum with two peaks at approximately 350 and 610 nm (Figure 4a) which are the typical λ_max_ of phycocyanin [24,49]. Previous studies indicate the possibility of this phycobiliprotein’s subunit separation in a HPLC-reversed-phase system [50,51].

**Figure 5:**
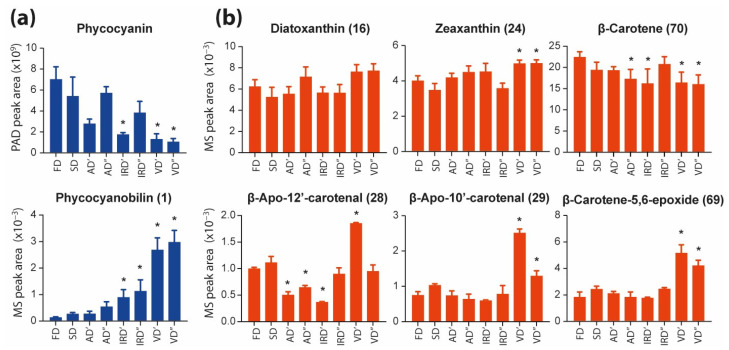
Changes in the content of phycocyanin and phycocyanobilin in aqueous extracts (**a**), the main carotenoids and their derivatives in ethanolic extracts (**b**) prepared from spirulina dried using different methods (FD, freeze drying; SD, sun drying; AD′, air drying at 40 °C; AD″, air drying at 75 °C; IRD′, infrared drying at 40 °C; IRD″, infrared drying at 75 °C; VD′, vacuum drying at 40 °C; VD″, vacuum drying at 75 °C). Data are presented as the mean ± SD (*n* = 3) of the PAD or the LC-MS peak intensity (10^7^). Values marked with asterisks are significantly different (Dunnett’s *t* test; *p* < 0.05) from those of the control sample (FD).

The C-phycocyanin is one of the three types of biliproteins that are part of phycobilisomes, and is extremely sensitive to heat and pH [51,52]. In the presented study, changes in the content of this compound in the aqueous extracts of dried spirulina were observed by comparing the areas under the peaks present in the chromatograms recorded by the PAD detector at 350 nm (Figure 5a, upper graph). The highest phycocyanin content was observed for the freeze-dried spirulina extracts. In extracts from spirulina dried at higher temperatures, this content was lower. The conventional drying of spirulina can reduce its phycocyanin content by up to 90% [12]. Choi and Lee (2018) [51] observed that the decrease in phycocyanin contents was most accelerated at a high temperature of 40 °C paired with light illumination. Similarly, Güroy et al. (2017) [53] reported a 35% lower phycocyanin content in oven-dried spirulina compared to freeze-dried spirulina. It can be expected that in the presented research, both the drying time and the temperature used had a major impact on the level of this compound. 

On the other hand, the analysis of the LC-MS data at a low mass range (<1200 Da) allowed for the tracking of the level of phycocyanobilin cleaved from phycocyanin. Previous studies have shown that phycocyanobilin can be cleaved from the apoprotein via several methods including acid hydrolysis, enzymatic cleavage, and solvolysis in methanol [54]. The results obtained in the presented research (Figure 5a, lower graph) indicate that a higher drying temperature, air movement, infrared light, and especially the use of a vacuum, favor the cleavage of phycocyanin, with the formation of phycocyanobilin.

The influence of pressure on the PCB cleavage efficiency was also observed by Roda-Serrat et al. (2018) [55], who reported that alcohol treatment under a high pressure and a high temperature efficiently cleaves the PCB from Lina Blue, which is a commercial phycocyanin powder used as a food additive. Similar conclusions were reached by Kamo et al. (2021) [56] when using a high pressure during the liquid extraction of Lina Blue phycobiliprotein powder, as well as cyanobacteria *Synechocystis*.

Chromatograms registered at 430 nm confirmed the presence of carotenoids and chlorophylls in both the aqueous (Figure 4a) and ethanolic extracts (Figure 4b). Due to the non-polar nature of these pigments, only the ethanolic extracts were considered for comparison. In the case of the carotenoids, the main pigments in the studied spirulina extracts were diatoxanthin (**16**), zeaxanthin (**24**) and *β*-carotene (**70**). In the case of diatoxanthin (**16**), no significant differences in its levels were observed in the ethanol extracts of spirulina dried using various methods (Figure 5b). On the other hand, the zeaxanthin (**24**) contents in the spirulina extracts were significantly higher after vacuum drying compared to those after freeze drying. Souza et al. (2022) observed a similar trend in the zeaxanthin concentrations in the chili pepper, which were higher in high-temperature drying treatments than in other treatments and reacted inversely with other yellow compounds, including *β*-carotene [57]. In the presented study, *β*-carotene (**70**) had the highest level in the extract from freeze-dried spirulina, while in spirulina extracts dried using other methods, the content of this compound was approximately 27–46% lower. A similar trend was observed by Stramarkou et al. (2021) [58]. In their studies, different methods of drying spirulina resulted in a decrease in *β*-carotene content of 32–84% compared to that of freeze-dried spirulina. The loss of *β*-carotene during plant processing results from the loss of tissue integrity, contact with oxygen, light and a high temperature. The main degradation products identified after food processing are isomers, oxidation and cleavage products [15,59]. Heat-induced changes in the stability of *β*-carotene result in its conversion into its cis-isomers, until an equilibrium state after prolonged heating is reached. However, the *β*-carotene isomerizes towards its cis form only if solubilized, and the extent of its isomerization is linearly dependent on the temperature being in the range of 100–140 °C [60]. The oxidation reaction products for carotenoids are quite numerous and differ in molecular weights. The initial oxidation products of *β*-carotene are epoxides. In the presented study, *β*-carotene-5, 6-epoxide (**69**) was detected, and its level, compared to that of the freeze-dried spirulina, was not significantly different in extracts of spirulina dried using other methods (Figure 5b). However, in spirulina extracts dried in a vacuum, especially at 40 °C, the content of this epoxide was twice as high. The highest contents in VD′ samples can be observed in the case of other *β*-carotene oxidation products, e.g., *β*-apo-12′-carotenal (**28**) and *β*-apo-10′-carotenal (**29**) (Figure 5b).

Another group of pigments that are sensitive to light, oxygen, heat, electromagnetic radiation, catalysts (metals), acids, enzyme activity and processing conditions are chlorophylls [61]. When comparing the water and ethanol extracts from spirulina, a higher concentration of chlorophylls and their derivatives can be found in ethanol extracts (Figure 4a,b). In the case of the aqueous extracts, the main compound from this group was chlorophyll *a* (**46**) (Figure 4a). However, the major compounds in the ethanolic extracts were the degradation products of chlorophylls, such as pheophytins. Solvents containing methanol, ethanol or 1-propanol are known to readily degrade chlorophyll *a* and chlorophyll *a* + *b* because the isomerization and allomerization of chlorophyll molecules occur very easily under acidic conditions, which should be avoided by neutralizing the solvent with magnesium carbonate [62]. 

**Figure 6:**
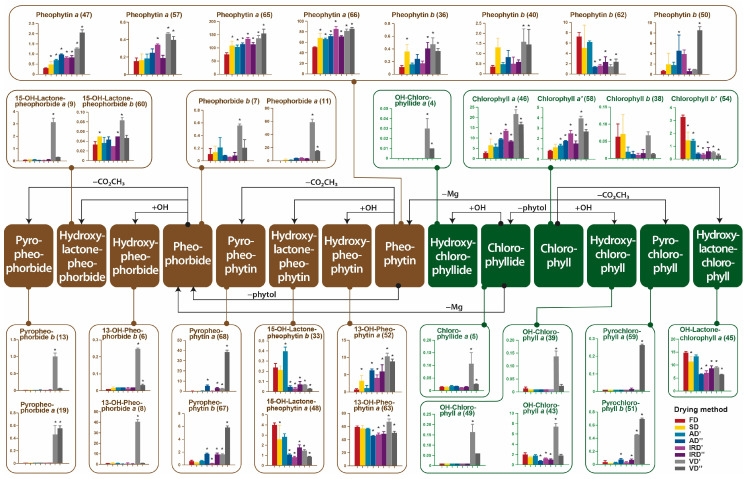
Mapping of chlorophylls’ degradation pathways and the dynamic variations of chlorophylls and derivatives in the ethanolic extracts prepared from spirulina dried using different methods (FD, freeze drying; SD, sun drying; AD′, air drying at 40 °C; AD″, air drying at 75 °C; IRD′, infrared drying at 40 °C; IRD″, infrared drying at 75 °C; VD′, vacuum drying at 40 °C; VD″, vacuum drying at 75 °C). Data are presented as the mean ± SD (*n* = 3) of mass spectrometric intensity (10^7^). Values marked with asterisks are significantly different (Dunnett’s *t* test; *p* < 0.05) from those of the control sample (FD). The compound numbers correspond to the numbers in Table S1.

Crude ethanolic extract from spirulina was used for metabolomic analyses, which allowed us to detect, among others, the degradation of chlorophylls to pheophytins, formed because of the removal of chelated magnesium. High concentrations of pheophytin *a* (**65**,**66**) were observed in these extracts (Figure 4b). Other studies also reported that the use of ethanol for extraction resulted in significant modifications to chlorophyll pigments [63]. Figure 6 illustrated the degradative pathway of the chlorophylls in the ethanolic extracts of spirulina dried using various methods. Various decomposition products showed discrepancies, probably resulting from enzymatic or non-enzymatic reactions dependent on the temperature, humidity and time of the drying processes. A trace level of chlorophyll *b* (*b*′) (**38**,**54**) was detected in the freeze-dried spirulina. Its content was significantly lower in spirulina dried at a higher temperature. In the case of chlorophyll *a* (*a*′) (**46**,**58**), an opposite trend was noticed, indicating its higher concentration in spirulina dried using methods other than freeze drying. Pheophytin *a* and *b* (**36**,**40**,**47**,**50**,**57**,**65**,**66**), in most cases, had the lowest content in the freeze-dried spirulina extract. Only for pheophytin *b* (**62**) were the changes during drying slightly different. The content of this compound, as well as that of some oxidized pheophytins (**48**,**63**), was the highest in the freeze-dried spirulina. Another group of compounds formed by the degradation of chlorophylls is that of pheophorbides *a*, *b* (**11**,**7**), which are the products of the demetallization and hydrolysis of phytol groups, which occurred at the highest level in spirulina vacuum-dried at 40 °C. The same trend was observed for their pyro-products (**13**,**19**), which were decomposition products after further demethoxycarbonylation, as well as their oxidation products, such as hydroxypheophorbide (**6**,**8**) and hydroxylactonepheophorbide (**9**,**60**). After the vacuum drying of spirulina at 40 °C, the formation of hydroxychlorophyll (**39**,**43**,**49**), chlorophyllide (**5**) and hydroxochlorophyllide (**4**) was observed. These results suggested that multiple chlorophyll decomposition reactions occurred during drying, including the loss of magnesium, the loss of the phytol chain under enzymatic or alkaline conditions, and demethoxycarbonylation and chlorophylls oxidization, probably due to chemical oxidation and/or the presence of enzymes in the spirulina tissue, including peroxidase, oxidase, and lipoxygenase. Therefore, the production of oxidized chlorophyll derivatives can be induced in both enzymatic and chemical reactions. The highest content of these compounds was observed in the biomass after the vacuum drying of spirulina at 40 °C. Under these conditions, it was observed that the pressure increased due to an increase in steam formation, which could not be removed by the pump quickly enough, and condensation occurred inside the dryer. Therefore, it is not recommended to work at maximum capacity because in these conditions, the vacuum system is not able to completely remove the generated vapors [64]. The condensation of water vapor in the chamber could have caused increased enzymatic activity, resulting in the degradation of native forms of chlorophylls. According to Roca et al. (2016) [65], mild heat processing, like steaming, induces the formation of C13^2^ epimers and pheophytins. Further thermal treatment increases the pheophytinization level and the formation of pyroderivatives. In fact, the difference in the intensity of chlorophylls’ degradation and the formation of pheophytin and pyropheophytin is a function of both heat exposure and the time of the process.

#### 2.2.2. The Impact of Drying Methods on Lipids

During microalgae processing and storage, lipids may undergo unfavorable enzymatic and non-enzymatic reactions, such as hydrolysis or oxidation. This can lead to a reduction in their nutritional value, cause off-flavors or even contribute to the formation of harmful substances. Previous studies have shown that lipolysis is mainly a problem during the wet stages of microalgae processing, such as harvesting or wet biomass storage [20,66]. Additionally, it was found that microalgal cell integrity is an important factor in the lipolytic stability of a wet biomass [67]. Therefore, a rapid reduction in the water content in a microalgae biomass after harvesting seems to be a key factor for limiting the lipid hydrolysis caused by endogenous lipolytic enzymes. However, when selecting a drying method, the focus should not only be on maximizing the reduction of lipolysis, but also the structural changes of the microalgal biomass (e.g., cell wall disruption), which could affect lipid extraction efficiency [68]. 

**Figure 7:**
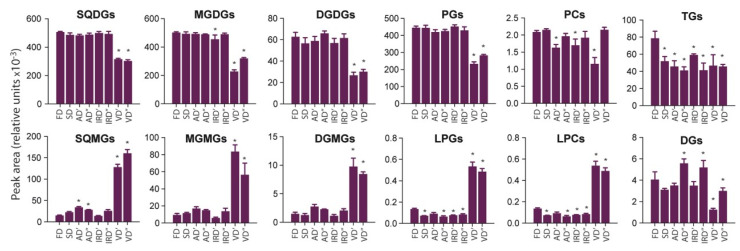
Changes in the content of selected lipid classes (TGs, triacylglycerols; DGs, diacylglycerols; SQDGs, sulfoquinovosyl diacylglycerols; SQMGs, sulfoquinovosyl monoacylglycerols; DGDGs, digalactosyl diacylglycerols; DGMGs, digalactosyl monoacylglycerols; MGDGs, monogalactosyl diacylglycerols; MGMGs, monogalactosyl monoacylglycerols; PGs, phosphatidylglycerols; LPGs, lysophosphatidylglycerols; PCs, phosphatidylcholines; LPCs, lysophosphatidylcholine) present in ethanolic extracts prepared from spirulina dried using different methods (FD, freeze drying; SD, sun drying; AD′, air drying at 40 °C; AD″, air drying at 75 °C; IRD′, infrared drying at 40 °C; IRD″, infrared drying at 75 °C; VD′, vacuum drying at 40 °C; VD″, vacuum drying at 75 °C). Data are presented as mean ± SD (*n* = 3) of mass spectrometric intensity (10^8^). Values marked with asterisks are significantly different (Dunnett’s *t* test; *p* < 0.05) from those of the control sample (FD).

Figure 7 shows the effect of *A. platensis* biomass drying methods on the sum of MS peak areas corresponding to all identified species belonging to a given lipid class. The highest breakdown of glycolipids, including SQDG, MGDG, and DGDG classes, was observed for the VD′ and VD″ methods. However, a greater degradation of SQDGs with a simultaneous increase in SQMG content was observed for vacuum drying at 75 °C compared to that of MGDGs and DGDGs, for which the difference between 40 and 75 °C was not-so-clearly visible. All the above-mentioned lipid classes can be hydrolyzed by enzymes with galactolipase activity; however, it should be noted that galactolipases can exhibit certain substrate preferences. For example, galactolipase from green microalgae *Chlorella kessleri* preferentially hydrolyzed DGDG species followed by MGDGs and SQDGs [69]. It can be surprising that the galactolipase activity in spirulina biomass towards SQDG species was higher during vacuuming at 75 than that at 40 °C. For example, the galactolipase from *C. kessleri* exhibits the highest activity at 37 °C, and above 40 °C, the activity dropped off rapidly [69]. To the best of our knowledge, there is no data in the literature on the enzymatic characterization of galactolipase from *A. platensis*; therefore, it is difficult to state that this enzyme will exhibit similar properties. Moreover, it must too be taken into account that the biomass cannot achieve the set temperature from the beginning of the process. After a warming-up period, the decrease in the moisture content leads to stabilization or to slowing down the increase in the biomass’s temperature, even though the temperature in the chamber is significantly higher [70]. Additionally, the reduced pressure in the vacuum chamber could cause microalgae cell disruption. On the other hand, the ratio of the sums of MS peak areas corresponding to SQMGs and SQDGs, respectively, indicated that the lower enzymatic hydrolysis of the SQDG class was observed for the F and I’ methods. A similar trend was observed for MGDG and DGDG species. However, in the cases of these classes, the S method was also characterized as being relatively lowly destructive.

The highest conversion of PLs to corresponding lysophospholipids (LPLs) was also observed for the VD′ and VD″ methods. These observations can indicate the highest enzymatic degradation of PG and PC species to LPG and LPC species, respectively, under conditions prevailing during vacuum drying. The reaction of the FA’s release from the structure of PL is typically catalyzed by phospholipase A_2_. In general, the enzyme plays a key role in lipid metabolism and is characterized by a high structural stability, owing to the presence of six disulfide bridges [71]. In turn, the lowest enzymatic degradation of PC and PG classes was observed for the FD, SD and IRD′ methods.

In the case of neutral lipids, a relatively low ratio of the sums of MS peak areas corresponding to TGs and DGs was observed for the VD′ and VD″ methods. This may indicate the lower enzymatic activity of the lipase present in *A. platensis*, especially if vacuum drying was carried out at 40 °C. The highest conversion of TGs to DGs was observed for the AD″ and IRD″ methods, in which the temperature of 75 °C was applied. The previous study on purified lipase from *Spirulina* spp. indicated that the enzyme was active in the temperature range of 37 to 60 °C, and no activity was observed at 70 °C [42]. However, this phenomenon, similar to that describing the conversion of SQDGs to SQMGs by galactolipase, may be related to the lower temperature of the biomass being dried, compared to the temperature in the drying chamber, especially in the initial phase of drying, as mentioned above. In comparison to other used drying methods, the FD method was characterized by a relatively high sum of MS peak areas corresponding to TG species. The reason behind this may be the higher degree of spirulina cell disruption in the biomass dried, when using the FD method and, consequently, the higher efficiency of TG extraction with ethanol.

#### 2.2.3. The Impact of Drying Methods on Polar Nitro-organic Compounds

**Figure 8:**
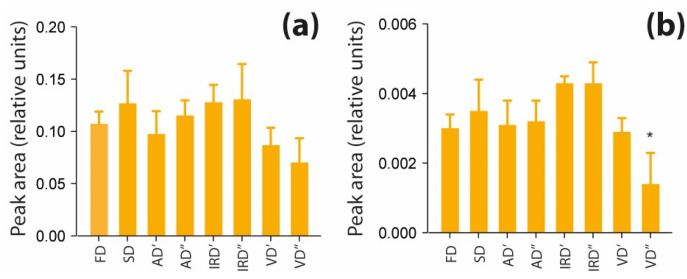
Changes in the contents of amino acids and dipeptides (**a**) and nucleobases (**b**) present in aqueous extracts prepared from spirulina dried using different methods (FD, freeze drying; SD, sun drying; AD′, air drying at 40 °C; AD″, air drying at 75 °C; IRD′, infrared drying at 40 °C; IRD″, infrared drying at 75 °C; V′, vacuum drying at 40 °C; V″, vacuum drying at 75 °C). Data are presented as mean ± SD (*n* = 3) of mass spectrometric intensity (10^8^). Values marked with asterisks are significantly different (Dunnett’s *t* test; *p* < 0.05) from those of the control sample (FD).

Amino acid profiles and contents differ among the used drying methods. Changes in amino acids’ compositions may result from their susceptibility to thermal hydrolysis or a Maillard reaction [72,73]. A bar graph presented in Figure 8a shows the combined peaks areas of amino acids and dipeptides (AAs and DPs) in the studied aqueous extracts of spirulina. This group of compounds seems to be affected the most by vacuum drying, but the difference in total peak areas of AAs and DPs compared to those in freeze-dried spirulina were not significant. Kharchuk et al. (2022) [17], who studied air drying’s impact on the chemical composition of spirulina, observed that the content of free nucleotides after the drying process decreased only slightly, while the content of DNA and RNA did not change. These results suggest that algal nucleic acids and their components remain stable in an air-drying process. In this study, the aqueous extraction of differently dried spirulina resulted in a similar recovery of nucleobases (Figure 8b). Although, subjecting spirulina to vacuum drying at 75 °C may have led to the degradation of these compounds, as in this particular case, the nucleobases levels are significantly lower than those obtained with freeze drying.

## 3. Materials and Methods

### 3.1. Strain and Growth Conditions

The strain of *A. platensis* (UTEX LB 2340) was obtained from the Centre for Environmental and Marine Studies, CESAM, Department of Chemistry, University of Aveiro, Aveiro, Portugal. It was cultured in glass bottles (2.5 L) on the Zarrouk medium [74] with continuous aeration. The illumination was provided using white fluorescent light (140 μmol/m^2^/s) with a photoperiod of a 12:12 h light and dark cycle. The temperature for the culture was maintained at 25 °C. Growth performance was monitored using a NanoDrop 2000c spectrophotometer (Thermo Scientific, Waltham, MA, USA) and the culture of *A. platensis* was harvested when the optical density at 560 nm reached 0.8. To recover the biomass, the suspension of the medium and the algae was vacuum-filtered using a paper filter (Ø = 35 mm) and then rinsed with distilled water to remove soluble salts. After filtration and washing, the filter papers with the collected wet biomass (3 mm) were divided into batches and stored at −20 °C until they were dried using various methods.

### 3.2. Drying Treatments

Five different drying methods were used: (a) freeze drying, (b) sun drying, (c) air drying, (d) infrared drying, and (e) vacuum drying. Samples were divided into equal batches and then dried in triplicate (8–12% residual moisture) using the procedures outlined below. All the lots were subsequently placed in the freezer at −18 °C before further steps.

(a) In freeze drying (FD), the batch of frozen spirulina paste was lyophilized in Christ Alpha 2–4 LSC freeze-dryer (Martin Christ Gefriertrocknungsanlagen GmbH, Osterode am Harz, Germany) at the temperature of −78 °C and a pressure of 0.94 mbar for 24 h.
(b) In sun drying (SD), samples were dried in an open space under direct sunlight at a temperature of 25–28 °C for 24 h.
(c) In air drying (AD), drying was performed in Florida Jerky air circulation oven (500 W, Klarestein, Berlin, Germany) at a temperature of 40 (AD′) or 75 °C (AD″) for 4 h.
(d) In infrared drying (IRD), drying was conducted at a temperature of 40 (IRD′) or 75 °C (IRD″) for 4 h in a Florida Jerky dehydrator (500 W, Klarestein, Berlin, Germany).
(e) In vacuum drying (VD), samples were dried in DZ-1BCII vacuum-drying oven (220 V, 800 W, ChemLand, Stargard, Poland) in two conditions: at a temperature of 40 °C (VD′) for 300 min or at 75 °C (VD″) for 240 min. The pressure inside the chamber in both tested conditions was reduced by 0.07–0.095 MPa compared to atmospheric pressure.

### 3.3. Extract Preparations

The dried spirulina powder was mixed with water or ethanol (50 mg/mL) and placed in an ultrasonic bath (POLSONIC, Warsaw, Poland) for 20 min at 25 °C. The samples were centrifuged (3000 rpm, 20 min) and clear supernatants were collected.

### 3.4. Q-Orbitrap HRMS Analysis

The aqueous and ethanolic extracts of spirulina (4 μL) were analyzed using the UltiMate 3000 UPLC system (Thermo Scientific Dionex, Bremen, Germany) coupled with a PDA detector and Q-Exactive^TM^ Focus quadrupole-Orbitrap mass spectrometer (Q-Exactive plus, Thermo Scientific, Bremen, Germany) equipped with a heated electrospray ionization source (HESI). LC separation was performed on a Kinetex XB-C18 column (150 × 4.6 mm, 5 μm, Phenomenex, Torrance, CA, USA) using a binary solvent system consisting of phase A (water, 0.1% formic acid, 10 mM of ammonium formate) and B (ACN:IPA = 2:8 *v*/*v*, 0.1% formic acid). A gradient elution was performed at a flow rate of 1 mL/min according to the following gradient program: 0 min using 100% phase A; 8 min using 30% phase B; 10 min using 70% phase B; 25 min using 100% phase B; 30 min using 100% phase B. And finally, the initial conditions were held for 5 min as a re-equilibration step. Absorbance spectra were recorded in PAD between 190 and 800 nm at a frequency of 0.5 Hz with a bandwidth of 4 nm while the chromatograms were monitored at 265, 350, 430 and 665 nm. The HESI parameters in negative and positive ion acquisition mode were as follows: auxiliary gas flow rate, 15 arb; sheath gas flow rate, 35 arb; sweep gas flow rate, 3 arb; capillary temperature, 350 °C; spray voltage, 2.5 kV; S-lens RF level, 50; and heater temperature, 300 °C. The full MS scan was set as follows: resolution, 70,000 FWHM; AGC target, 2 × 10^5^; max inject time, 100 ms; and scan range, 120–1200 *m*/*z*. The data-dependent MS2 parameters were as follows: resolution, 17,500 FWHM; isolation window, 3.0 *m*/*z*; collision energy, 30 eV; AGC target, 2 × 10^5^; max inject time, 100 ms. Data were analyzed using Xcalibur 4.1 software (Thermo Fisher Scientific, Waltham, MA, USA).

### 3.5. Data Processing

Raw data files were processed using Compound Discoverer software (v. 3.3, Thermo Scientific, San Jose, CA, USA). The identification of compounds was based on their having a highly accurate mass (mass tolerance of 5 ppm), retention time (RT) behavior (RT tolerance of 0.2 min) as well as their fragmentation pattern and its comparison with the MS/MS spectra of compounds available in online libraries (*m*/*z* Cloud, ChemSpider, Human Metabolome database—https://hmdb.ca/, Mass Bank—https://massbank.eu/MassBank/ (both accessed on 12 October 2023)) or using local customized databases of different classes of phytochemicals created based on data in the literature implemented in Compound Discover 3.3 software. Additionally, two local mass list databases were searched consisting of 4400 endogenous metabolites, extractables and leachables with a mass tolerance the same as above. MS data were normalized using the total sum normalization (TSN) method. The total abundance of a metabolite class was calculated by summing up all normalized areas of compounds belonging to the class. The PLS-DA and HCA of 18 metabolite classes after the drying of spirulina were performed using MetaboAnalyst 5.0 (https://www.metaboanalyst.ca/ (accessed on 15 November 2023)). The parameters used for HCA were as follows: distance measure method—Euclidean; clustering method—Ward. The graphical illustration of chromatograms, the global composition of spirulina extracts and the statistical analysis of changes in the content of pigments, lipids and polar nitro-organic compounds were performed using GraphPad Prism 10.0.2.

## 4. Conclusions

From the results of this study, it is clear that the choice of drying method can significantly influence the qualitative and quantitative composition of metabolites in a final, dried spirulina product. The differentiation analyses indicated that the metabolomic and lipidomic profiles of the spirulina biomass were influenced the least by air drying at a lower temperature (AD′), when compared to the control (FD). Moreover, a clear difference was shown between vacuum drying and other studied drying methods. These two methods (VD′ and VD″) had a negative effect on almost all investigated metabolite classes, resulting in either a low level of metabolites in their native form, or a high level of their degradation products. This was most likely due to steam condensation caused by insufficient water removal from the drying chamber in the vacuum dryer. This issue did not occur in other methods, which could have resulted in the limitation of some enzymatic degradation processes requiring the presence of water. In the case of freeze drying, a low temperature prevents the loss of heat-sensitive compounds; however, ice crystals formed during the freezing stage cause the breaking of the cell walls and, in consequence, increase the release of metabolites from the cells. In this study, the freeze drying of spirulina was the most suitable method to retain secondary metabolites, such as phycocyanin and carotenoid pigments. For the other classes of compounds, there were no such visibly favorable methods. In the case of the chlorophyll pigments, the extraction solvent was the main source of modifications, but the vacuum drying resulted in the presence of a higher level of degradation products compared to other drying methods. Vacuum drying also had the greatest impact on the lipid profiles, leading to extensive degradation. The polar nitro-organic compounds’ levels were similar across the different drying methods used, again with the exception of the vacuum-drying methods, where corresponding peak areas were significantly lower. This study demonstrates the usefulness of omics technique implementation as a strategy for characterizing the drying processes of microalgae. The proposed workflow could be useful for future research, as although it has been proven that freeze drying preserves nutrients and bioactives in microalgae, it is still necessary to seek more affordable and high-throughput drying methods.

## Data Availability

Data are included in the article and Supplementary Materials.

## Supplementary Materials

The following supporting information can be downloaded at: https://www.mdpi.com/article/10.3390/molecules29081747/s1. Table S1: Retention time (Rt, min), proposed formula, theoretical *m*/*z* value of the precursor ion (Da), experimental *m*/*z* value of the precursor ion (Da), accuracy (Δm, ppm), *m*/*z* values of diagnostic fragment ions (Da) of compounds identified as pigments in spirulina extracts with the use of LC-Q-Orbitrap HRMS in positive ion mode; Table S2: Retention time (Rt, min), proposed formula, theoretical *m*/*z* value of the precursor ion (Da), experimental *m*/*z* value of the precursor ion (Da), accuracy (Δm, ppm), *m*/*z* values of diagnostic fragment ions (Da) of compounds identified as sulfoquinovosyl diacylglycerols (SQDGs) and sulfoquinovosyl monoacylglycerols (SQMGs) in ethanolic spirulina extracts with the use of LC-Q-Orbitrap HRMS in negative ion mode; Table S3: Retention time (Rt, min), proposed formula, theoretical *m*/*z* value of the precursor ion (Da), experimental *m*/*z* value of the precursor ion (Da), accuracy (Δm, ppm), *m*/*z* values of diagnostic fragment ions (Da) of compounds identified as monogalactosyl diacylglycerol (MGDGs), monogalactosyl monoacylglycerol (MGMGs), digalactosyl diacylglycerols (DGDGs) and digalactosyl monoacylglycerols (DGMGs) in ethanolic spirulina extracts with the use of LC-Q-Orbitrap HRMS in positive ion mode; Table S4: Retention time (Rt, min), proposed formula, theoretical *m*/*z* value of the precursor ion (Da), experimental *m*/*z* value of the precursor ion (Da), accuracy (Δm, ppm), *m*/*z* values of diagnostic fragment ions (Da) of compounds identified as phosphatidylglycerols (PGs), and lysophosphatidylglycerols (LPGs) in ethanolic spirulina extracts with the use of LC-Q-Orbitrap HRMS in negative ion mode; Table S5: Retention time (Rt, min), proposed formula, theoretical *m*/*z* value of the precursor ion (Da), experimental *m*/*z* value of the precursor ion (Da), accuracy (Δm, ppm), *m*/*z* values of diagnostic fragment ions (Da) of compounds identified as phosphatidylcholines (PCs), lysophosphatidylcholines (LPCs) and phosphatidylethanolamines (PEs) in ethanolic spirulina extracts with the use of LC-Q-Orbitrap HRMS in positive ion mode; Table S6: Retention time (Rt, min), proposed formula, theoretical *m*/*z* value of the precursor ion (Da), experimental *m*/*z* value of the precursor ion (Da), accuracy (Δm, ppm), *m*/*z* values of diagnostic fragment ions (Da) of compounds identified as ceramide phosphoinositols (PI-Cers) in ethanolic spirulina extracts with the use of LC-Q-Orbitrap HRMS in negative ion mode; Table S7: Retention time (Rt, min), proposed formula, theoretical mass of the parent ion (Da), experimental mass of the parent ion (Da), accuracy (Δm, ppm), *m*/*z* values of diagnostic mass fragments (Da) of compounds tentatively identified as triacylglycerols (TGs) and diacylglycerols (DGs) in spirulina extracts with the use of LC-Q-Orbitrap HRMS in positive ion mode; Table S8: Retention time (Rt, min), proposed formula, theoretical *m*/*z* value of the precursor ion (Da), experimental *m*/*z* value of the precursor ion (Da), accuracy (Δm, ppm), *m*/*z* values of diagnostic fragment ions (Da) of compounds identified as amino acids, peptides and other nitrogen compounds in aqueous spirulina extracts with the use of LC-Q-Orbitrap HRMS in positive ion mode.
