# Supplementary material for: The Impact of Different Drying Methods on the Metabolomic and Lipidomic Profiles of Arthrospira platensis
Source: Molecules. 2024 Apr 12;29(8):1747. doi: 10.3390/molecules29081747 (PMC11051859; doi:10.3390/molecules29081747)
Supplement: Supplementary file 1 [file molecules-29-01747-s001.zip › molecules-2906773-supplementary.pdf]

The Impact of Different Drying Methods on the Metabolomic and Lipidomic Profiles of *Arthrospira platensis*

Marika Mróz [1], Karol Parchem [1,2], Joanna Jóźwik [1], M. Rosário Domingues [3,4] and Barbara Kusznierewicz [1,*]

[1] Department of Chemistry, Technology and Biotechnology of Food, Faculty of Chemistry, Gdańsk University of Technology, 11/12 Narutowicza St., 80-233 Gdańsk, Poland; marika.mroz@pg.edu.pl (M.M.); karol.parchem@pg.edu.pl (K.P.); joanna.jozwik@pg.edu.pl (J.J.)

[2] Department of Analytical Chemistry, Faculty of Chemical Technology, University of Pardubice, Studentská 573, 532 10 Pardubice, Czech Republic

[3] Mass Spectrometry Centre, LAQV-REQUIMTE, Department of Chemistry, University of Aveiro, Santiago University Campus, 3810-193 Aveiro, Portugal; mrd@ua.pt

[4] Centre for Environmental and Marine Studies, CESAM, Department of Chemistry, University of Aveiro, Santiago University Campus, 3810-193 Aveiro, Portugal

* Correspondence: barbara.kusznierewicz@pg.edu.pl

Supplementary materials

Table S1. Retention time (Rt, min), proposed formula, theoretical *m*/*z* value of the precursor ion (Da), experimental *m*/*z* value of the precursor ion (Da), accuracy (Δm, ppm), *m*/*z* values of diagnostic fragment ions (Da) of compounds identified as pigments in spirulina extracts with the use of LC-Q-Orbitrap HRMS in positive ion mode.

No	Rt [min]	Compound	Formula	Theoretical $[M+H]^+$	Experimental $[M+H]^+$	Δm [ppm]	MS/MS	Cl
1	11.80	Phycocyanobilin (I)	$C_{33}H_{38}N_4O_6$	587.28696	587.28626	-0.27	587.286; 299.139; 464.218	P
2	13.03	15^1-OH-Lactone-pheophorbide *a* (I)	$C_{35}H_{36}N_4O_7$	625.26623	625.26546	0.30	607.255; 547.234	Ch
3	13.31	13^2-OH-Pheophorbide *a* (I)	$C_{35}H_{36}N_4O_6$	609.27131	609.27054	-0.36	591.260; 519.237; 549.250	Ch
4	13.49	13^2-OH-Chlorophyllide *a*	$C_{35}H_{34}MgN_4O_6$	631.24071	631.23963	-0.82	-	Ch
5	13.54	Chlorophyllide *a*	$C_{35}H_{34}MgN_4O_5$	615.24579	615.24487	0.60	-	Ch
6	13.56	13^2-OH-Pheophorbide *b*	$C_{35}H_{34}N_4O_7$	623.25058	623.24983	0.30	-	Ch
7	14.06	Pheophorbide *b*	$C_{35}H_{34}N_4O_6$	607.25566	607.25494	0.30	547.234; 591.260	Ch
8	14.17	13^2-OH-Pheophorbide *a* (II)	$C_{35}H_{36}N_4O_6$	609.27131	609.27050	-0.43	591.259; 531.239; 559.234; 577.245; 549.251	Ch
9	14.31	15^1-OH-Lactone-pheophorbide *a* (II)	$C_{35}H_{36}N_4O_7$	625.26623	625.26552	0.20	538.257; 565.244; 581.275; 566.249;	Ch
10	14.53	Adonirubin	$C_{40}H_{52}O_3$	581.39947	581.39902	0.16	119.085; 145.101; 147.117; 563.387; 467.371	C
11	14.67	Pheophorbide *a*	$C_{35}H_{36}N_4O_5$	593.27640	593.27561	-0.38	533.255; 565.282	Ch
12	14.77	Chlorophyll *c*	$C_{35}H_{32}N_4O_5$	589.24510	589.24440	-0.24	529.224; 588.409; 591.257; 485.232; 277.215	Ch
13	15.21	Pyro-pheophorbide *b*	$C_{33}H_{32}N_4O_4$	549.25018	549.24961	-0.01	521.254; 503.244; 476.220	Ch
14	15.24	Diadinoxanthin (I)	$C_{40}H_{54}O_3$	583.41512	583.41459	0.02	221.154; 165.0912; 119.086; 145.101; 565.401; 491.401	C
15	15.38	Myxoxanthophyll	$C_{46}H_{66}O_7$	731.48868	731.48580	-1.48	119.086; 145.101; 175.148; 71.049; 135.116; 525.372; 713.500	C
16	15.39	Diatoxanthin (I)	$C_{40}H_{54}O_2$	567.42021	567.41967	0.02	119.086; 145.101; 133.101; 549.418; 475.359	C
17	15.81	Phycocyanobilin (II)	$C_{33}H_{38}N_4O_6$	587.28696	587.28652	0.32	587.286; 299.139; 464.218	P
18	15.84	Antheraxanthin (I)	$C_{40}H_{56}O_3$	585.43077	585.43022	-0.01	119.086; 175.148; 145.101; 133.101; 135.117; 567.426; 475.355	C
19	15.87	Pyro-pheophorbide *a*	$C_{33}H_{34}N_4O_3$	535.27091	535.27032	-0.10	184.073; 136.062; 507.277; 190.159; 435.252;	Ch
20	16.36	Adonixanthin	$C_{40}H_{54}O_3$	583.41512	583.41451	-0.13	147.117; 119.086; 173.133; 145.101; 135.117; 565.401	C
21	16.85	Antheraxanthin (II)	$C_{40}H_{56}O_3$	585.43077	585.43023	0.01	119.086; 175.148; 145.101; 133.101; 135.117; 567.426; 475.355	C
22	16.99	Diatoxanthin (II)	$C_{40}H_{54}O_2$	567.42020	567.41969	0.07	119.086; 145.101; 133.101; 549.418	C
23	17.34	Diadinoxanthin (II)	$C_{40}H_{54}O_3$	583.41512	583.41441	-0.20	119.086; 145.101; 135.117; 121.101; 175.148; 203.143; 565.401	C
24	17.34	Zeaxanthine	$C_{40}H_{56}O_2$	569.43586	569.43555	-0.54	119.086; 175.148; 135.117; 145.101; 477.366; 551.422	C
25	17.35	Vaucheriaxanthin	$C_{40}H_{56}O_5$	617.42060	617.41986	-0.40	121.101; 119.086; 145.101; 135.117, 599.409; 581.400; 557.399	C
26	17.35	Antheraxanthin (III)	$C_{40}H_{56}O_3$	585.43077	585.43007	-0.23	119.086; 145.101; 175.148; 135.117; 567.426; 549.412; 475.355	C
27	18.28	3-OH-Echinenone (I)	$C_{40}H_{54}O_2$	567.42020	567.41960	-0.09	203.143; 133.065; 119.086; 145.101; 175.148; 549.410	C
28	18.29	β-Apo-12′-carotenal	$C_{25}H_{34}O$	351.26879	351.26889	0.28	119.086; 105.070; 93.070; 69.071; 95.086; 107.086	
29	18.73	β-Apo-10′-carotenal	$C_{27}H_{36}O$	377.28444	377.28384	-0.15	119.086; 95.086; 105.070; 93.070; 81.070; 69.070; 133.101	C
30	18.91	Antheraxanthin (IV)	$C_{40}H_{56}O_3$	585.43077	585.43005	-0.22	145.101; 119.086; 175.148; 135.117; 567.426; 549.412; 475.355	C
31	19.02	Canthaxanthin	$C_{40}H_{52}O_2$	565.40455	565.40397	-0.07	203.143; 133.065; 365.662; 204.146; 119.086; 215.143; 548.417	C
32	19.11	3-OH-Echinenone (II)	$C_{40}H_{54}O_2$	567.42020	567.41967	0.02	133.065; 203.143; 119.086; 145.101; 175.148; 549.410	C
33	19.61	15^1-OH-Lactone pheophytin *b* (I)	$C_{55}H_{72}N_4O_8$	917.54284	917.54207	-0.24	639.245; 313.273; 873.552; 579.225	Ch

34	20.49	β-Apo-8′-karotenal	$C_{30}H_{40}O$	417.31574	417.31511	-0.19	119.086; 95.086; 107.086; 121.101; 105.070; 93.070; 69.070	C
35	20.51	15^1-OH-Lactone-pheophytin *a* (I)	$C_{55}H_{74}N_4O_7$	903.56358	903.56263	-0.58	625.265; 885.549; 576.269	Ch
36	21.31	Pheophytin *b* (I)	$C_{55}H_{72}N_4O_6$	885.55301	885.55214	-0.37	593.275; 533.254; 867.545; 607.252	Ch
37	21.37	Chlorophyll *b*	$C_{55}H_{70}MgN_4O_6$	907.52241	907.52119	-0.73	-	Ch
38	21.82	13^2-OH-Pheophytin *a* (I)	$C_{55}H_{74}N_4O_6$	887.56866	887.56735	-0.86	609.271; 313.274; 593.276; 549.249	Ch
39	22.09	OH-Chlorophyll *a* (I)	$C_{55}H_{72}MgN_4O_6$	909.53806	909.53694	-0.76	613.229; 891.527; 631.229	Ch
40	22.30	Pheophytin *b* (II)	$C_{55}H_{72}N_4O_6$	885.55301	885.55220	-0.30	575.266; 607.255; 867.540	Ch
41	22.60	β-Cryptoxathin-5'.6'-epoxide	$C_{40}H_{56}O_2$	569.43585	569.43526	-0.08	205.159; 109.101; 121.101; 119.086; 145.101; 551.421	C
42	22.66	13^2-OH-Pheophytin *a* (II)	$C_{55}H_{74}N_4O_6$	887.56866	887.56764	-0.53	591.259; 609.270; 869.557	Ch
43	22.67	OH-Chlorophyll *a* (II)	$C_{55}H_{72}MgN_4O_6$	909.53806	909.53712	-0.44	613.229; 631.238; 891.525; 892.529	Ch
44	22.70	Divinyl chlorophyll *a* (I)	$C_{55}H_{70}MgN_4O_5$	891.52749	891.52645	-0.55	613.229; 553.207; 581.203	Ch
45	22.80	15^1-OH-Lactone chlorophyll *a*	$C_{55}H_{72}MgN_4O_7$	925.53297	925.53216	-0.30	647.235; 838.524; 602.238; 560.226	Ch
46	23.02	Chlorophyll *a*	$C_{55}H_{72}MgN_4O_5$	893.54314	893.54223	-0.42	615.244; 555.224; 583.219; 833.523	Ch
47	23.03	Pheophytin *a* (I)	$C_{55}H_{74}N_4O_5$	871.57375	871.57302	-0.20	593.275; 533.255; 576.265	Ch
48	23.10	15^1-OH-Lactone-pheophytin *a* (II)	$C_{55}H_{74}N_4O_7$	903.56358	903.56242	-0.68	625.265; 607.255; 885.552	Ch
49	23.12	OH-Chlorophyll *a* (III)	$C_{55}H_{72}MgN_4O_6$	909.53806	909.53633	-1.30	631.239; 613.229; 553.207; 598.201	Ch
50	23.16	Pheophytin *b* (III)	$C_{55}H_{72}N_4O_6$	885.55301	885.55193	-0.60	607.254; 503.245; 592.264	Ch
51	23.17	Pyro-chlorophyll *b*	$C_{53}H_{68}MgN_4O_4$	849.51693	849.51601	0.40	571.219; 525.209; 821.520; 543.224	Ch
52	23.20	13^2-OH-Pheophytin *a* (III)	$C_{55}H_{74}N_4O_6$	887.56866	887.56739	-0.79	591.259; 609.270	Ch
53	23.20	13^2-OH-Pheophytin *b*	$C_{55}H_{72}N_4O_7$	901.54793	901.54707	-0.24	623.250; 519.239; 563.229; 605.239; 536.241	Ch
54	23.30	Chlorophyll *b*'	$C_{55}H_{70}MgN_4O_6$	907.52241	907.52094	-0.20	629.224; 586.229; 631.225; 628.236	Ch
55	23.45	Divinyl chlorophyll *a* (II)	$C_{55}H_{70}MgN_4O_5$	891.52749	891.52577	-1.28	613.228; 553.208; 631.239; 571.218	Ch
56	23.53	Echinenone	$C_{40}H_{54}O$	551.42529	551.42464	-0.19	133.065; 203.143; 119.086; 121.101; 69.070; 185.133; 177.164	C
57	23.57	Pheophytin *a* (II)	$C_{55}H_{74}N_4O_5$	871.57375	871.57297	-0.27	593.275; 533.254	Ch
58	23.58	Chlorophyll *a*'	$C_{55}H_{72}MgN_4O_5$	893.54314	893.54212	-0.56	615.243; 555.223; 583.217; 833.520	Ch
59	23.61	Pyro-chlorophyll *a* (I)	$C_{53}H_{70}MgN_4O_3$	835.53766	835.53630	1.00	557.239; 313.273; 529.248; 575.503	Ch
60	23.82	15^1-OH-Lactone-pheophorbide *b*	$C_{35}H_{34}N_4O_8$	639.24549	639.24471	0.40	-	Ch
61	23.84	15^1-OH-Lactone pheophytin *b* (II)	$C_{55}H_{72}N_4O_8$	917.54284	917.54135	-0.71	639.245; 623.249; 589.208; 887.532	Ch
62	24.14	Pheophytin *b* (IV)	$C_{55}H_{72}N_4O_6$	885.55301	885.55160	-0.60	607.254; 503.244; 591.259	Ch
63	24.29	13^2-OH-Pheophytin *a* (IV)	$C_{55}H_{74}N_4O_6$	887.56866	887.56784	-0.32	591.259; 609.271; 869.557; 531.237	Ch
64	24.46	15^1-OH-Lactone-pheophytin *a* (III)	$C_{55}H_{74}N_4O_7$	903.56358	903.56272	-0.33	625.265; 885.553; 537.249; 607.255	Ch
65	24.81	Pheophytin *a* (III)	$C_{55}H_{74}N_4O_5$	871.57375	871.57285	-0.41	593.275; 533.254; 636.837	Ch
66	25.12	Pheophytin *a* (IV)	$C_{55}H_{74}N_4O_5$	871.57375	871.57297	-0.27	593.276; 533.254; 429.751	Ch
67	25.43	Pyro-pheophytin *b*	$C_{53}H_{70}N_4O_4$	827.54753	827.54682	-0.21	549.249; 503.243; 521.254; 799.549; 548.246	Ch
68	25.91	Pyro-pheophytin *a*	$C_{53}H_{72}N_4O_3$	813.56827	813.56732	-0.49	535.269; 203.526	Ch
69	26.34	β-Carotene-5.6-epoxide	$C_{40}H_{56}O$	553.44094	553.44039	0.13	119.086; 121.101; 145.101; 348.281; 177.164; 147.117; 535.426	C
70	26.40	β-Carotene	$C_{40}H_{56}$	536.43820	536.43763*	-1.07	119.086; 177.164; 121.101; 171.117; 444.375; 147.117;	C
71	28.66	Phytoene	$C_{40}H_{64}$	545.50862	545.50819	0.20	81.070; 95.086; 109.101; 123.117; 69.071; 121.101; 149.133	C

Classes: C, carotenoids and derivatives; Ch, chlorophylls and derivatives; P, phycocyanin. * Molecular radical ion $[M]^{+\bullet}$.

Table S2. Retention time (Rt, min), proposed formula, theoretical *m/z* value of the precursor ion (Da), experimental *m/z* value of the precursor ion (Da), accuracy (Δm, ppm), *m/z* values of diagnostic fragment ions (Da) of compounds identified as sulfoquinovosyl diacylglycerols (SQDGs) and sulfoquinovosyl monoacylglycerols (SQMGs) in ethanolic spirulina extracts with the use of LC-Q-Orbitrap HRMS in negative ion mode.

No	Rt [min]	Compound	Formula	Theoretical $[M-H]^-$	Experimental $[M-H]^-$	Δm [ppm]	MS/MS
72	12.23	SQMG(18:3)	$C_{27}H_{46}O_{11}S$	577.2683	577.2695	2.03	225.007; 277.218;
73	12.48	SQMG(18:2)	$C_{27}H_{48}O_{11}S$	579.2839	579.2849	1.75	225.007; 279.232
74	12.7	SQMG(16:0)	$C_{25}H_{48}O_{11}S$	555.2839	555.2848	1.62	225.007
75	12.82	SQMG(18:1)	$C_{27}H_{50}O_{11}S$	581.2996	581.3004	1.38	225.007
76	13.37	SQMG(18:0)	$C_{27}H_{52}O_{11}S$	583.3152	583.3162	1.71	225.007
77	16.42	SQDG(30:1)	$C_{39}H_{72}O_{12}S$	763.4666	763.4678	1.54	255.233; 225.007;
78	16.76	SQDG(32:2)	$C_{41}H_{74}O_{12}S$	789.4822	789.4833	1.36	225.007
79	16.9	SQDG(34:3)	$C_{43}H_{76}O_{12}S$	815.4979	815.4988	1.1	255.233
80	17.18	SQDG(36:4)	$C_{45}H_{78}O_{12}S$	841.5136	841.5139	0.36	225.006
81	17.22	SQDG(34:3)	$C_{43}H_{76}O_{12}S$	815.4979	815.4987	0.98	255.233
82	17.24	SQDG(30:0)	$C_{39}H_{74}O_{12}S$	765.4823	765.483	0.88	225.008
83	17.44	SQDG(32:1)	$C_{41}H_{76}O_{12}S$	791.4979	791.4988	1.11	225.007
84	17.66	SQDG(34:2)	$C_{43}H_{78}O_{12}S$	817.5136	817.5145	1.1	225.008
85	18.07	SQDG(36:3)	$C_{45}H_{80}O_{12}S$	843.5292	843.5301	1.07	255.233; 225.008
86	18.34	SQDG(32:0)	$C_{41}H_{78}O_{12}S$	793.5136	793.5143	1.09	.225.007
87	18.51	SQDG(34:1)	$C_{43}H_{80}O_{12}S$	819.5292	819.5303	1.34	225.007
88	18.72	SQDG(36:2)	$C_{45}H_{82}O_{12}S$	845.5449	845.5456	0.83	225.006
89	19.44	SQDG(34:0)	$C_{43}H_{82}O_{12}S$	821.5449	821.5453	0.49	225.007
90	19.57	SQDG(36:1)	$C_{45}H_{84}O_{12}S$	847.5605	847.5606	0.06	225.007
91	20.47	SQDG(36:0)	$C_{45}H_{86}O_{12}S$	849.5762	849.5769	0.82	225.007

Table S3. Retention time (Rt, min), proposed formula, theoretical *m*/*z* value of the precursor ion (Da), experimental *m*/*z* value of the precursor ion (Da), accuracy (Δm, ppm), m/z values of diagnostic fragment ions (Da) of compounds identified as monogalactosyl diacylglycerol (MGDGs), monogalactosyl monoacylglycerol (MGMGs), digalactosyl diacylglycerols (DGDGs) and digalactosyl monoacylglycerols (DGMGs) in ethanolic spirulina extracts with the use of LC-Q-Orbitrap HRMS in positive ion mode.

No	Rt [min]	Compound - Possible fatty acids	Formula	Theoretical $[M+NH_4]^+$	Experimental $[M+NH_4]^+$	Δm [ppm]	MS/MS
92	13	MGMG(16:2)	$C_{25}H_{44}O_9$	506.3329	506.3322	-1.41	309.242
93	13.06	DGMG(16:1)	$C_{31}H_{56}O_{14}$	670.4014	670.4003	-1.69	311.258
94	13.23	DGMG(18:2)	$C_{33}H_{58}O_{14}$	696.417	696.4164	-0.8	337.274
95	13.47	DGMG(16:0) isomer 1	$C_{31}H_{58}O_{14}$	672.417	672.4163	-1.1	313.273
96	13.48	MGMG(16:1)	$C_{25}H_{46}O_9$	508.3486	508.3479	-1.44	311.258
97	13.67	DGMG(16:0) isomer 2	$C_{31}H_{58}O_{14}$	672.417	672.4164	-0.92	313.273
98	13.9	MGMG(18:2)	$C_{27}H_{48}O_9$	534.3642	534.3638	-0.81	337.274
99	14.12	MGMG(16:0)	$C_{25}H_{48}O_9$	510.3642	510.3638	-0.84	313.274
100	14.22	MGMG(18:1)	$C_{27}H_{50}O_9$	536.3799	536.3795	-0.83	339.289
101	14.44	MGMG(18:1)	$C_{27}H_{50}O_9$	536.3799	536.3792	-1.28	339.289
102	15.32	MGMG(18:0)	$C_{27}H_{52}O_9$	538.3955	538.3952	-0.55	341.305
103	18.11	DGDG(34:5)	$C_{49}H_{82}O_{15}$	928.5997	928.5986	-1.18	309.242; 335.258; 569.456; 311.258
104	18.39	MGDG(34:6)	$C_{43}H_{70}O_{10}$	764.5313	764.5307	-0.78	307.227; 309.242; 335.258; 333.242; 567.440; 379.284
105	18.74	DGDG(36:6)	$C_{51}H_{84}O_{15}$	954.6154	954.6171	1.74	335.258; 595.473
106	18.84	MGDG(32:4)	$C_{41}H_{70}O_{10}$	740.5313	740.5305	-1.06	311.258; 309.242; 543.441; 335.258; 307.227
107	19	DGDG(34:4)	$C_{49}H_{84}O_{15}$	930.6154	930.615	-0.43	311.258; 335.258; 571.472
108	19.19	MGDG(34:5)	$C_{43}H_{72}O_{10}$	766.5469	766.546	-1.15	309.242; 335.258; 569.456; 311.258
109	19.34	DGDG(36:5)	$C_{51}H_{86}O_{15}$	956.631	956.6309	-0.08	337.274; 335.258; 597.488
110	19.45	MGDG(33:4)	$C_{42}H_{72}O_{10}$	754.5469	754.5463	-0.76	311.258; 321.242; 557.457
111	19.5	DGDG(32:2)	$C_{47}H_{84}O_{15}$	906.6154	906.6144	-1.06	313.274; 547.472; 309.242; 311.258
112	19.54	MGDG(32:3)	$C_{41}H_{72}O_{10}$	742.5469	742.5468	-0.12	311.258; 309.242; 545.457
113	19.67	DGDG(34:3)	$C_{49}H_{86}O_{15}$	932.631	932.6301	-0.94	573.487; 311.258; 337.273
114	19.74	MGDG(36:6)	$C_{45}H_{74}O_{10}$	792.5626	792.5626	0.03	335.258; 595.472; 337.274
115	19.78	MGDG(35:5)	$C_{44}H_{74}O_{10}$	780.5626	780.5619	-0.91	323.258; 335.258; 583.472; 337.273
116	19.83	MGDG(32:3)	$C_{41}H_{72}O_{10}$	742.5469	742.5468	-0.12	285.243; 545.457; 311.258; 309.242; 335.258
117	19.95	DGDG(36:4)	$C_{51}H_{88}O_{15}$	958.6467	958.6465	-0.16	337.274; 599.504; 339.289; 335.258;
118	20.06	MGDG(34:4)	$C_{43}H_{74}O_{10}$	768.5626	768.5621	-0.61	311.258; 335.258; 571.472
119	20.22	MGDG(33:3)	$C_{42}H_{74}O_{10}$	756.5626	756.562	-0.86	311.258; 559.472; 323.258
120	20.37	DGDG(32:1)	$C_{47}H_{86}O_{15}$	908.631	908.6307	-0.29	549.488; 313.274; 311.258
121	20.37	MGDG(36:5)	$C_{45}H_{76}O_{10}$	794.5782	794.5775	-0.94	337.274; 335.258; 597.488
122	20.53	MGDG(33:3)	$C_{42}H_{74}O_{10}$	756.5626	756.5621	-0.7	313.273; 321.242; 559.472
123	20.58	MGDG(32:2)	$C_{41}H_{74}O_{10}$	744.5626	744.5624	-0.3	547.472; 311.258; 313.274; 285.242; 309.243; 337.274
124	20.64	MGDG(35:4)	$C_{44}H_{76}O_{10}$	782.5782	782.5775	-0.88	325.274; 335.258; 585.488; 311.258
125	20.67	DGDG(34:2)	$C_{49}H_{88}O_{15}$	934.6467	934.6455	-1.27	313.274; 575.503; 337.273
126	20.75	MGDG(34:3)	$C_{43}H_{76}O_{10}$	770.5782	770.5779	-0.33	311.258; 573.488; 337.274
127	20.85	MGDG(32:1)	$C_{41}H_{76}O_{10}$	746.5782	746.5781	-0.18	313.273; 549.487; 311.258
128	20.95	DGDG(36:3)	$C_{51}H_{90}O_{15}$	960.6623	960.6613	-1.04	313.274; 601.519; 363.289
129	20.96	MGDG(36:4)	$C_{45}H_{78}O_{10}$	796.5939	796.593	-1.18	337.274; 599.503; 311.258; 313.274; 335.258; 363.289
130	21.09	MGDG(34:3)	$C_{43}H_{76}O_{10}$	770.5782	770.578	-0.26	313.274; 335.258; 573.488
131	21.19	MGDG(33:2)	$C_{42}H_{76}O_{10}$	758.5782	758.5781	-0.1	313.274; 561.488; 323.258
132	21.24	MGDG(36:4)	$C_{45}H_{78}O_{10}$	796.5939	796.5935	-0.5	339.289; 335.258; 599.504; 311.258; 363.289; 337.274
133	21.31	MGDG(35:3)	$C_{44}H_{78}O_{10}$	784.5939	784.5934	-0.58	587.504; 337.274; 311.258; 351.290; 327.289
134	21.37	DGDG(32:0)	$C_{47}H_{88}O_{15}$	910.6467	910.6464	-0.37	313.274; 551.504
135	21.41	MGDG(32:1)	$C_{41}H_{76}O_{10}$	746.5782	746.5773	-1.24	313.274; 549.488; 311.258; 337.273
136	21.49	DGDG(34:1)	$C_{49}H_{90}O_{15}$	936.6623	936.662	-0.32	577.519; 313.274; 339.290
137	21.69	MGDG(34:2)	$C_{43}H_{78}O_{10}$	772.5939	772.5933	-0.75	313.274; 575.503; 337.274
138	21.74	DGDG(36:2)	$C_{51}H_{92}O_{15}$	962.678	962.6766	-1.48	313.274; 603.534; 365.305
139	21.98	MGDG(33:1)	$C_{42}H_{78}O_{10}$	760.5939	760.5933	-0.84	313.273; 563.503; 325.274
140	22.17	MGDG(35:2)	$C_{44}H_{80}O_{10}$	786.6095	786.6094	-0.16	589.519; 313.274; 351.289; 311.258
141	22.36	MGDG(32:0)	$C_{41}H_{78}O_{10}$	748.5939	748.5937	-0.28	313.274; 551.504
142	22.49	MGDG(34:1)	$C_{43}H_{80}O_{10}$	774.6095	774.609	-0.63	313.274; 577.519; 339.289
143	22.65	MGDG(36:2)	$C_{45}H_{82}O_{10}$	800.6252	800.6249	-0.4	313.274; 603.535; 365.305; 341.305; 337.274
144	22.99	MGDG(35:1)	$C_{44}H_{82}O_{10}$	788.6252	788.6246	-0.72	313.273; 591.535; 353.305; 327.289
145	23.41	MGDG(34:0)	$C_{43}H_{82}O_{10}$	776.6252	776.6238	-1.75	579.534; 313.273; 341.305
146	23.48	MGDG(36:1)	$C_{45}H_{84}O_{10}$	802.6408	802.6396	-1.43	605.550; 341.305; 339.289; 313.273; 367.320
147	24.23	MGDG(36:0)	$C_{45}H_{86}O_{10}$	804.6565	804.6562	-0.39	607.566; 313.274; 369.336; 341.305

Table S4. Retention time (Rt, min), proposed formula, theoretical *m/z* value of the precursor ion (Da), experimental *m/z* value of the precursor ion (Da), accuracy (Δm, ppm), *m/z* values of diagnostic fragment ions (Da) of compounds identified as phosphatidylglycerols (PGs), and lysophosphatidylglycerols (LPGs) in ethanolic spirulina extracts with the use of LC-Q-Orbitrap HRMS in negative ion mode.

No	Rt [min]	Compound	Formula	Theoretical $[M-H]^-$	Experimental $[M-H]^-$	Δm [ppm]	MS/MS
148	12.5	LPG(18:3)	$C_{24}H_{43}O_9P$	505.2566	505.2575	1.86	277.217; 152.994; 227.032; 245.042; 171.005
149	12.77	LPG(18:2)	$C_{24}H_{45}O_9P$	507.2723	507.273	1.4	279.233; 152.9951; 227.0321; 245.043; 171.005
150	13.08	LPG(16:0) isomer 1	$C_{22}H_{45}O_9P$	483.2723	483.2722	-0.3	255.232; 152.994; 227.032; 171.006; 245.044
151	13.23	LPG(18:1)	$C_{24}H_{47}O_9P$	509.2879	509.2876	-0.65	281.248; 152.994; 227.032; 245.042
152	13.83	LPG(16:0) isomer 2	$C_{22}H_{45}O_9P$	483.2723	483.2728	0.96	255.233; 152.995; 227.032
153	13.94	LPG(18:0)	$C_{24}H_{49}O_9P$	511.3036	511.3037	0.16	283.264; 152.994; 227.032; 171.005
154	17.74	PG(36:5)	$C_{42}H_{73}O_{10}P$	767.4863	767.4869	0.83	277.217; 279.233; 152.995
155	17.93	PG(32:2)	$C_{38}H_{71}O_{10}P$	717.4707	717.4703	-0.51	255.232; 251.201; 253.217; 152.994
156	18.41	PG(36:4)	$C_{42}H_{75}O_{10}P$	769.502	769.5032	1.6	279.233; 251.202; 253.217; 277.217; 489.260; 152.995
157	18.68	PG(32:2)	$C_{38}H_{71}O_{10}P$	717.4707	717.4716	1.2	255.233; 227.201; 251.201; 152.995; 279.233; 253.217
158	18.75	PG(34:3)	$C_{40}H_{73}O_{10}P$	743.4863	743.4873	1.35	253.217; 279.233; 152.995; 507.273; 489.265
159	19.09	PG(36:4)	$C_{42}H_{75}O_{10}P$	769.502	769.5063	5.57	279.233; 251.201; 253.217; 152.995
160	19.15	PG(34:2)	$C_{40}H_{75}O_{10}P$	745.502	745.503	1.33	279.233; 255.233; 152.995; 489.262; 465.262
161	19.32	PG(33:2)	$C_{39}H_{73}O_{10}P$	731.4863	731.4874	1.46	255.232; 152.995; 493.257
162	19.4	PG(35:3)	$C_{41}H_{75}O_{10}P$	757.502	757.5029	1.15	267.233; 279.233; 507.270; 253.218
163	19.66	PG(32:0)	$C_{38}H_{75}O_{10}P$	721.502	721.5013	-0.1	255.232; 152.994; 465.260; 483.273
164	19.75	PG(34:2)	$C_{40}H_{75}O_{10}P$	745.502	745.503	1.33	255.233; 279.233; 152.995; 507.272; 489.262
165	19.88	PG(32:0)	$C_{38}H_{75}O_{10}P$	721.502	721.5014	-0.83	255.232; 465.261; 152.994; 483.275
166	20.31	PG(33:1)	$C_{39}H_{75}O_{10}P$	733.502	733.5035	2.02	255.233; 267.233; 152.995; 495.274
167	20.31	PG(36:3)	$C_{42}H_{77}O_{10}P$	771.5176	771.5184	1	255.233; 281.249; 152.995; 279.233; 251.201
168	20.67	PG(34:2)	$C_{40}H_{75}O_{10}P$	745.502	745.5032	1.57	255.233; 279.233; 152.995; 507.272; 489.262
169	20.69	PG(35:2)	$C_{41}H_{77}O_{10}P$	759.5176	759.5189	1.74	269.249; 255.233; 279.233; 152.995; 293.249
170	20.81	PG(32:0)	$C_{38}H_{75}O_{10}P$	721.502	721.5015	-0.66	255.232; 152.995; 465.262; 483.272; 171.005
171	20.97	PG(34:1)	$C_{40}H_{77}O_{10}P$	747.5176	747.5189	1.77	255.233; 281.249; 152.995; 491.277
172	21.02	PG(36:2)	$C_{42}H_{79}O_{10}P$	773.5333	773.5367	4.45	281.249; 255.233; 152.995; 253.217; 509.292; 491.277
173	21.03	PG(32:0)	$C_{38}H_{75}O_{10}P$	721.502	721.5037	2.301	255.233; 152.995; 465.262; 483.272; 171.005
174	21.27	PG(34:1)	$C_{40}H_{77}O_{10}P$	747.5176	747.5183	0.95	255.233; 281.248; 152.995; 509.287
175	21.31	PG(36:2)	$C_{42}H_{79}O_{10}P$	773.5333	773.5345	1.53	255.233; 281.249; 307.264; 152.995; 283.264
176	21.72	PG(35:1)	$C_{41}H_{79}O_{10}P$	761.5333	761.5347	1.8	255.233; 295.264; 152.995; 269.248
177	22.2	PG(34:0)	$C_{40}H_{79}O_{10}P$	749.5333	749.5321	-1.59	255.233; 283.264; 152.995; 493.295
178	22.45	PG(34:0)	$C_{40}H_{79}O_{10}P$	749.5333	749.5344	1.42	255.232; 283.264; 152.995; 511.304; 493.293
179	23.95	PG(36:0)	$C_{42}H_{83}O_{10}P$	777.5646	777.5656	1.22	255.233; 311.296; 152.995; 283.263; 539.336
180	25.99	PG(34:2)	$C_{40}H_{75}O_{10}P$	745.502	745.5034	1.82	255.233; 279.233; 152.995; 507.273; 489.261

Table S5. Retention time (Rt, min), proposed formula, theoretical *m/z* value of the precursor ion (Da), experimental *m/z* value of the precursor ion (Da), accuracy (Δm, ppm), *m/z* values of diagnostic fragment ions (Da) of compounds identified as phosphatidylcholines (PCs), lysophosphatidylcholines (LPCs) and phosphatidylethanolamines (PEs) in ethanolic spirulina extracts with the use of LC-Q-Orbitrap HRMS in positive ion mode.

No	Rt [min]	Compound	Formula	Theoretical $[M+H]^+$	Experimental $[M+H]^+$	Δm [ppm]	MS/MS
181	14.58	LPC(18:1)	$C_{26}H_{52}NO_7P$	522.356	522.3555	-1.02	184.073
182	21.19	PC(34:2)	$C_{42}H_{80}NO_8P$	758.57	758.5781	10.73	184.073
183	21.7	PC(36:2)	$C_{44}H_{84}NO_8P$	786.6013	786.6083	8.89	184.073
184	22.47	PC(37:2)	$C_{45}H_{86}NO_8P$	800.6169	800.6235	8.29	184.073
185	22.86	PE(36:2)	$C_{41}H_{78}NO_8P$	744.5543	744.5538	-0.63	603.534

Table S6. Retention time (Rt, min), proposed formula, theoretical *m/z* value of the precursor ion (Da), experimental *m/z* value of the precursor ion (Da), accuracy (Δm, ppm), *m/z* values of diagnostic fragment ions (Da) of compounds identified as ceramide phosphoinositols (PI-Cers) in ethanolic spirulina extracts with the use of LC-Q-Orbitrap HRMS in negative ion mode.

No	Rt [min]	Compound	Formula	Theoretical $[M-H]^-$	Experimental $[M-H]^-$	Δm [ppm]	MS/MS
186	17.12	PI-Cer(t18:0/16:0)	$C_{40}H_{80}NO_{12}P$	796.534	796.5329	-1.38	241.011; 259.021
187	17.38	PI-Cer(t19:0/16:0)	$C_{41}H_{82}NO_{12}P$	810.5496	810.5483	-1.63	241.011; 259.021
188	17.84	PI-Cer(t18:0/18:0)	$C_{42}H_{84}NO_{12}P$	824.5653	824.5635	-2.21	241.011; 259.022

Table S7. Retention time (Rt, min), proposed formula, theoretical mass of the parent ion (Da), experimental mass of the parent ion (Da), accuracy (Δm, ppm), m/z values of diagnostic mass fragments (Da) of compounds tentatively identified as triacylglycerols (TGs) and diacylglycerols (DGs) in spirulina extracts with the use of LC-Q-Orbitrap HRMS in positive ion mode.

No	Rt [min]	Compound - Possible fatty acids	Formula	Theoretical $[M+NH_4]^+$	Experimental $[M+NH_4]^+$	Δm [ppm]	MS/MS
189	18.21	DG(34:2)	$C_{37}H_{68}O_5$	610.541	610.5404	-0.97	313.274; 337.273
190	20.16	DG(34:4)	$C_{37}H_{64}O_5$	606.5097	606.509	-1.2	311.258; 335.258
191	21.13	DG(34:3)	$C_{37}H_{66}O_5$	608.5254	608.5243	-1.82	313.274; 335.258
192	21.69	DG(34:2)	$C_{37}H_{68}O_5$	610.541	610.5406	-0.67	313.274; 337.274
193	22.28	DG(34:3)	$C_{37}H_{66}O_5$	608.5254	608.5247	-1.12	311.258; 313.273; 337.273; 335.258
194	22.59	DG(34:3)	$C_{37}H_{66}O_5$	608.5254	608.5246	-1.32	313.274; 335.258
195	23.16	DG(34:2)	$C_{37}H_{68}O_5$	610.541	610.54	-1.57	313.273; 337.273
196	23.52	TG(34:1)	$C_{37}H_{68}O_6$	626.536	626.5356	-0.57	327.253; 521.457; 409.331; 353.268; 381.300; 355.284; 328.256; 493.425
197	23.73	DG(32:0)	$C_{35}H_{68}O_5$	586.541	586.5406	-0.7	313.274
198	23.86	DG(34:1)	$C_{37}H_{70}O_5$	612.5567	612.5561	-0.99	313.273; 339.289
199	24.3	TG(34:0)	$C_{37}H_{70}O_6$	628.5516	628.551	-0.91	355.284; 383.315; 523.472; 327.253; 411.347
200	24.35	TG(36:1)	$C_{39}H_{72}O_6$	654.5673	654.5667	-0.9	355.284; 409.331; 549.488; 381.300; 521.456; 383.316; 437.362
201	24.59	TG(38:2)	$C_{41}H_{74}O_6$	680.5829	680.582	-1.28	381.300; 575.503; 383.316; 407.316; 409.331
202	24.66	TG(34:0)	$C_{37}H_{70}O_6$	628.5516	628.5514	-0.33	355.284; 383.315; 523.472; 411.347; 327.253; 495.441
203	24.98	TG(34:0)	$C_{37}H_{70}O_6$	628.5516	628.5511	-0.72	355.284; 383.315; 523.472; 411.347; 495.441; 327.253
204	25.01	TG(36:0)	$C_{39}H_{74}O_6$	656.5829	656.5825	-0.67	383.315; 551.504; 355.284; 411.347; 523.472
205	25.19	TG(40:2)	$C_{43}H_{78}O_6$	708.6142	708.6133	-1.21	409.331; 603.535; 575.503; 411.347; 435.347; 437.363
206	25.34	TG(36:0)	$C_{39}H_{74}O_6$	656.5829	656.5826	-0.49	383.315; 551.504; 411.347; 355.284; 523.472
207	25.62	TG(40:1)	$C_{43}H_{80}O_6$	710.6299	710.6296	-0.45	411.347; 437.363; 577.519; 465.394; 409.331; 439.378
208	25.67	TG(42:2)	$C_{45}H_{82}O_6$	736.6455	736.645	-0.65	437.363; 603.535; 491.410; 439.379; 549.488; 575.504; 465.394
209	26.16	TG(44:2)	$C_{47}H_{86}O_6$	764.6768	764.6762	-0.77	465.394; 575.503; 491.409; 603.534; 467.411; 577.518; 493.426
210	26.17	TG(42:1)	$C_{45}H_{84}O_6$	738.6612	738.6602	-1.33	439.378; 465.394; 577.519; 549.487
211	26.18	TG(40:0)	$C_{43}H_{82}O_6$	712.6455	712.6447	-1.19	439.378; 411.347; 467.410; 579.534; 523.472
212	26.22	TG(38:1)	$C_{41}H_{76}O_6$	682.5986	682.5975	-1.65	383.315; 409.331; 577.519; 411.347; 549.488; 437.363
213	26.22	TG(40:1)	$C_{43}H_{80}O_6$	710.6299	710.6288	-1.48	411.347; 437.362; 409.331; 577.519; 439.378; 605.549
214	26.23	TG(38:0)	$C_{41}H_{78}O_6$	684.6142	684.6135	-1.07	411.347; 383.315; 551.503; 579.534; 439.380; 495.442; 467.416
215	26.26	TG(36:0)	$C_{39}H_{74}O_6$	656.5829	656.5823	-0.86	383.316; 551.503; 411.347; 355.284; 523.472
216	26.56	TG(42:0)	$C_{45}H_{86}O_6$	740.6768	740.6761	-0.96	467.410; 495.441; 523.472; 551.503; 439.379
217	26.57	TG(44:1)	$C_{47}H_{88}O_6$	766.6925	766.6916	-1.19	467.410; 521.456; 493.426; 549.488; 577.519
218	26.58	TG(46:2)	$C_{49}H_{90}O_6$	792.7081	792.7072	-1.19	493.426; 603.535; 547.472; 549.488; 521.457; 575.504
219	26.66	TG(48:3)	$C_{51}H_{92}O_6$	818.7238	818.7225	-1.55	519.441; 521.457; 547.472; 601.518; 545.456; 575.503; 573.488
220	26.67	TG(50:4)	$C_{53}H_{94}O_6$	844.7394	844.7392	-0.24	547.472; 575.503; 573.488; 571.472; 599.503
221	26.74	TG(54:6)	$C_{57}H_{98}O_6$	896.7707	896.7699	-0.89	599.503; 551.503; 577.519; 601.518; 597.488
222	26.77	TG(52:5)	$C_{55}H_{96}O_6$	870.7551	870.7545	-0.67	575.503; 573.487; 597.487
223	26.97	TG(46:1)	$C_{49}H_{92}O_6$	794.7238	794.7231	-0.82	495.441; 521.456; 549.487; 577.519; 493.425; 605.550; 523.472
224	26.98	TG(48:2)	$C_{51}H_{94}O_6$	820.7394	820.7386	-0.99	521.457; 549.488; 603.535; 547.472; 575.503; 577.519;
225	26.98	TG(44:0)	$C_{47}H_{90}O_6$	768.7081	768.7074	-0.91	495.441; 523.472; 551.503; 467.412; 509.457; 579.532
226	27.01	TG(50:3)	$C_{53}H_{96}O_6$	846.7551	846.754	-1.34	575.503; 549.487; 547.472; 573.488; 601.519; 603.533
227	27.03	TG(54:5)	$C_{57}H_{100}O_6$	898.7864	898.7843	-2.33	599.503; 601.519; 551.503
228	27.04	TG(52:4)	$C_{55}H_{98}O_6$	872.7707	872.7702	-0.57	575.503; 599.503; 573.487; 601.518
229	27.35	TG(48:1)	$C_{51}H_{96}O_6$	822.7551	822.7539	-1.45	523.472; 549.487; 577.519; 521.456; 551.503; 605.550
230	27.35	TG(50:2)	$C_{53}H_{98}O_6$	848.7707	848.7695	-1.45	549.487; 603.534; 575.503; 577.519; 551.502; 563.501
231	27.38	TG(46:0)	$C_{49}H_{94}O_6$	796.7394	796.7386	-0.94	523.472; 551.503; 495.440; 579.534; 537.488
232	27.38	TG(52:3)	$C_{55}H_{100}O_6$	874.7864	874.7855	-1	575.503; 603.534; 577.518; 601.518
233	27.38	TG(54:4)	$C_{57}H_{102}O_6$	900.802	900.8015	-0.61	601.518; 603.534; 599.503; 605.550
234	27.72	TG(52:2)	$C_{55}H_{102}O_6$	876.802	876.8011	-0.97	577.519; 603.535; 575.503; 579.535; 605.549
235	27.73	TG(48:1)	$C_{51}H_{96}O_6$	822.7551	822.754	-1.38	523.472; 549.488; 577.519; 551.503; 521.457; 579.534
236	27.73	TG(50:1)	$C_{53}H_{100}O_6$	850.7864	850.7855	-1.1	577.519; 551.503; 549.488; 605.550; 579.533
237	27.73	TG(54:3)	$C_{57}H_{104}O_6$	902.8177	902.8167	-1.09	603.535
238	27.75	TG(48:0)	$C_{51}H_{98}O_6$	824.7707	824.7696	-1.34	551.503; 523.472; 579.534; 607.566; 565.519
239	28.05	TG(56:3)	$C_{59}H_{108}O_6$	930.849	930.8497	0.72	631.566; 603.534; 633.582; 601.518; 629.554
240	28.06	TG(54:2)	$C_{57}H_{106}O_6$	904.8333	904.8323	-1.06	605.550; 603.534; 607.565; 631.565; 577.520
241	28.07	TG(52:1)	$C_{55}H_{104}O_6$	878.8177	878.8166	-1.26	579.534; 577.519; 605.550; 607.564
242	28.1	TG(50:1)	$C_{53}H_{100}O_6$	850.7864	850.7854	-1.17	577.519; 551.503; 549.488; 605.549; 579.533
243	28.11	TG(50:0)	$C_{53}H_{102}O_6$	852.802	852.801	-1.14	579.535; 551.503; 607.565; 565.519; 523.471
244	28.44	TG(56:2)	$C_{59}H_{110}O_6$	932.8646	932.8632	-1.48	633.581; 577.519; 603.534; 659.597; 605.550; 631.566
245	28.46	TG(54:1)	$C_{57}H_{108}O_6$	906.849	906.8478	-1.35	605.550; 607.564; 633.581; 577.519
246	28.48	TG(52:0)	$C_{55}H_{106}O_6$	880.8333	880.8328	-0.61	579.535; 607.565; 551.503
247	28.48	TG(52:1)	$C_{55}H_{104}O_6$	878.8177	878.8159	-2.03	579.534; 577.519; 605.550; 607.565; 551.503
248	28.48	TG(52:2)	$C_{55}H_{102}O_6$	876.802	876.8008	-1.32	577.519; 603.535; 575.503; 579.533; 605.549

249	28.87	TG(56:1)	$C_{59}H_{112}O_6$	934.8803	934.879	-1.42	635.597; 661.612; 577.519; 633.582; 605.550
250	28.9	TG(54:0)	$C_{57}H_{110}O_6$	908.8646	908.8634	-1.32	607.565; 635.597; 609.572; 579.534; 551.503
251	29.61	TG(56:0)	$C_{59}H_{114}O_6$	936.8959	936.8953	-0.61	663.628; 635.597; 551.503; 579.535; 607.566
252	29.74	TG(58:1)	$C_{61}H_{116}O_6$	962.9116	962.9108	-0.86	663.628; 577.519; 689.644; 661.612; 963.915
253	29.94	TG(58:0)	$C_{61}H_{118}O_6$	964.9272	964.9265	-0.77	663.628; 691.660; 579.535; 607.566

Table S8. Retention time (Rt, min), proposed formula, theoretical *m*/*z* value of the precursor ion (Da), experimental *m*/*z* value of the precursor ion (Da), accuracy (Δm, ppm), *m*/*z* values of diagnostic fragment ions (Da) of compounds identified as amino acids, peptides and other nitrogen compounds in aqueous spirulina extracts with the use of LC-Q-Orbitrap HRMS in positive ion mode.

No	Rt [min]	Compound	Formula	Theoretical $[M+H]^+$	Experimental $[M+H]^+$	Δm [ppm]	MS/MS	Reference
254	1.57	Spermidine	$C_7H_{19}N_3$	146.1657	146.1652	0.22	72.082; 84.0815; 58.066; 112.112	MSBNK-Keio_Univ-KO003974
255	1.64	Lys	$C_6H_{14}N_2O_2$	147.1133	147.1128	0.16	84.081; 55.055	MSBNK-Antwerp_Univ-METOX_P100301_FB57
256	1.67	Ala-Lys	$C_9H_{19}N_3O_3$	218.1505	218.1499	-0.12	84.081; 56.050; 67.055; 130.086	MSBNK-Keio_Univ-KO002291
257	1.69	His	$C_6H_9N_3O_2$	156.0773	156.0769	0.56	83.061; 93.045; 110.072; 82.053; 68.050; 95.061	MSBNK-BGC_Munich-RP002003
258	1.70	Lys(me2)	$C_8H_{18}N_2O_2$	175.1446	175.1441	0.11	70.066; 84.081; 72.081; 56.050; 130.098; 68.050	HMDB0341183
259	1.72	Agmatine	$C_5H_{14}N_4$	131.1297	131.1292	0.83	72.082; 60.056; 55.055; 97.076	MSBNK-Keio_Univ-KO002256
260	1.73	Gln	$C_5H_{10}N_2O_3$	147.0769	147.0765	0.41	84.045; 56.050	MSBNK-IPB_Halle-PB000468
261	1.74	Arg	$C_6H_{14}N_4O_2$	175.1195	175.1190	0.19	70.066; 60.056; 71.050; 72.081	MSBNK-IPB_Halle-PB000421
262	1.76	Glu	$C_5H_9NO_4$	148.0610	148.0604	0.11	84.045; 56.050	MSBNK-BGC_Munich-RP001403
263	1.77	Ser-A**rg**	$C_9H_{19}N_5O_4$	262.1515	262.1509	-0.48	70.066; 60.045; 116.071; 60.056; 158.092; 175.119; 72.082	HMDB0029033
264	1.78	Arg-Asn	$C_{10}H_{20}N_6O_4$	289.1624	289.1617	-0.60	70.065; 115.087; 60.056	HMDB0028704
265	1.79	Arg-Gly	$C_8H_{17}N_5O_3$	232.1410	232.1404	-0.30	70.066; 116.071; 60.056; 112.087; 72.081; 115.087; 100.051	HMDB0028709
266	1.84	Methionine sulfoxide	$C_5H_{11}NO_3S$	166.0538	166.0533	0.16	56.050; 74.024; 75.027; 102.055	HMDB0002005
267	1.95	Gln-Ala	$C_8H_{15}N_3O_4$	218.1141	218.1135	-0.25	84.045; 83.061; 56.050; 101.071	HMDB0028790
268	2.00	N8-Acetylspermidine	$C_9H_{21}N_3O$	188.1763	188.1757	-0.08	84.081; 58.066; 72.081; 114.092;	HMDB0002189
269	2.14	Val-His	$C_{11}H_{18}N_4O_3$	255.1457	255.145	-0.77	110.072; 72.0815; 57.034; 156.077; 55.055	HMDB0029129
270	2.22	Thr-Gln	$C_9H_{17}N_3O_5$	248.1246	248.124	-0.37	84.045; 74.061; 56.050; 60.056; 114.103	HMDB0029059
271	2.27	Cytidine diphosphate	$C_9H_{15}N_3O_{11}P_2$	404.0260	404.0253	-0.36	112.051; 97.029	HMDB0001546
272	2.31	Asn-Val	$C_9H_{17}N_3O_4$	232.1297	232.1291	-0.82	72.081; 55.055; 141.000; 70.066; 87.056	HMDB0028744
273	2.36	L-Ergothioneine	$C_9H_{15}N_3O_2S$	230.0963	230.0959	0.21	127.033; 69.058; 60.082; 68.050; 100.022; 67.042	MSBNK-MSSJ-MSJ00864
274	2.39	Val-Lys	$C_{11}H_{23}N_3O_3$	246.1818	246.1811	-0.40	72.081; 130.086; 55.055; 129.102; 147.113	HMDB0029132
275	2.49	His-Pro	$C_{11}H_{16}N_4O_3$	253.1301	253.1294	-0.55	110.072; 83.061; 93.045; 70.066; 116.071; 156.078	HMDB0028893
276	2.57	Arg-Val	$C_{11}H_{23}N_5O_3$	274.1879	274.1873	-0.42	72.082; 70.066; 60.056; 175.119; 55.055; 112.087	HMDB0028722
277	2.59	Pro-Gly	$C_7H_{12}N_2O_3$	173.0926	173.0921	0.30	70.066; 68.050; 116.071; 53.003	HMDB0011178
278	2.82	Gln-Val	$C_{10}H_{19}N_3O_4$	246.1454	246.1448	-0.39	72.082; 130.050; 55.055	HMDB0028832
279	2.86	Met	$C_5H_{11}NO_2S$	150.0589	150.0584	0.55	74.024; 87.027; 102.055	MSBNK-BGC_Munich-RP001902
280	3.14	Lys-Leu	$C_{12}H_{25}N_3O_3$	260.1974	260.1968	-0.30	84.081; 86.097; 129.102	HMDB0028955
281	3.20	L-Glutathione (reduced)	$C_{10}H_{17}N_3O_6S$	308.0916	308.091	-0.23	116.017; 162.022	MSBNK-NAIST-KNA00122
282	3.21	His-Leu	$C_{12}H_{20}N_4O_3$	269.1614	269.1607	-0.37	110.072; 156.077; 95.061; 69.071; 83.061; 93.045	HMDB0028889
283	3.33	Met-Arg	$C_{11}H_{23}N_5O_3S$	306.1599	306.1593	-0.37	104.053; 56.050; 60.056; 116.071; 61.011; 112.087	HMDB0028967
284	3.34	Leu-Gly	$C_8H_{16}N_2O_3$	189.1239	189.1234	-0.01	55.055; 57.058; 70.066	HMDB0028929
285	3.50	Niacin (vit. B3)	$C_6H_5NO_2$	124.0399	124.0395	1.57	80.050; 53.039; 124.039; 52.019; 97.048	HMDB0001488
286	3.53	Ala-Pro	$C_8H_{14}N_2O_3$	187.1083	187.1077	-0.1	70.066; 116.071; 55.055; 84.045	HMDB0028695
287	3.60	Cytidine	$C_9H_{13}N_3O_5$	244.0933	244.0927	-0.31	112.051	HMDB0000089
288	3.61	Gln-Pro	$C_{10}H_{17}N_3O_4$	244.1297	244.1291	-0.27	70.066; 84.045; 83.061; 116.071	HMDB0028805
289	3.77	Guanine	$C_5H_5N_5O$	152.0572	152.0568	0.28	110.035; 55.030; 107.036; 135.030	MSBNK-Antwerp_Univ-METOX_P100706_EF88
290	3.83	Arg-Ile	$C_{12}H_{25}N_5O_3$	288.2035	288.2029	-0.35	86.097; 70.066; 60.056; 69.071; 112.087; 72.082	HMDB0028712
291	3.83	Leu/Ile	$C_6H_{13}NO_2$	132.1024	132.102	0.75	53.003; 86.097; 55.055; 69.071; 57.058; 56.050	MSBNK-IPB_Halle-PB000398
292	3.93	Ser-Met	$C_8H_{16}N_2O_4S$	237.0909	237.0903	-0.23	60.045; 56.050; 104.053; 61.011; 133.032; 102.055; 84.045; 87.027	HMDB0029045
293	3.96	Hpro-Arg	$C_{11}H_{21}N_5O_4$	288.1672	288.1666	-0.17	70.066; 175.119; 116.071; 60.056; 158.092; 86.061	HMDB0028857
294	4.02	Adenine	$C_5H_5N_5$	136.0623	136.0617	-0.46	119.035	MSBNK-RIKEN-PR100020
295	4.04	γ-Glu-Val	$C_{10}H_{18}N_2O_5$	247.1294	247.1288	-0.15	84.045; 72.081; 55.055; 56.050; 130.050; 118.086	HMDB0011172
296	4.04	Lys-Phe	$C_{15}H_{23}N_3O_3$	294.1818	294.1812	-1.35	84.0814; 120.081; 129.102; 56.050	HMDB0028958
297	4.05	Guanosine monophosphate	$C_{10}H_{14}N_5O_8P$	364.0658	364.0652	-0.16	152.057	HMDB0001397
298	4.07	Hypoxanthine	$C_5H_4N_4O$	137.0463	137.0458	0.37	55.030; 110.035; 82.041; 137.046; 94.041; 67.030	HMDB0014581
299	4.16	Val-Met	$C_{10}H_{20}N_2O_3S$	249.1273	249.1267	-0.11	72.082; 55.055; 56.050; 104.053; 61.011; 57.058	HMDB0259742
300	4.17	Phe	$C_9H_{11}NO_2$	166.0868	166.0863	0.45	103.055; 120.081; 91.055; 95.050; 79.055; 93.070	MSBNK-BGC_Munich-RP000403
301	4.25	2'-Deoxyadenosine	$C_{10}H_{13}N_5O_3$	252.1096	252.1091	-0.15	136.062; 73.029; 99.044	HMDB0000101
302	4.50	Gln-Leu	$C_{11}H_{21}N_3O_4$	260.1610	260.1605	-0.05	84.045; 86.097; 83.061; 56.050; 132.102; 101.071; 129.066	HMDB0028801
303	4.57	Ala-Leu	$C_9H_{18}N_2O_3$	203.1396	203.1391	0.37	86.097; 132.102; 69.071; 72.081	HMDB0028691
304	4.58	Thr-Leu	$C_{10}H_{20}N_2O_4$	233.1501	233.1495	-0.23	74.061; 56.050; 86.097; 57.034; 58.066	HMDB0259044
305	4.75	Leu-Pro	$C_{11}H_{20}N_2O_3$	229.1552	229.1547	0.05	70.066; 86.097; 69.071	HMDB0011175

306	4.88	Phe-Gln	$C_{14}H_{19}N_3O_4$	294.1454	294.1448	-0.25	120.081; 84.045; 166.086; 56.050	HMDB0028993
307	4.92	Phe-Gly	$C_{11}H_{14}N_2O_3$	223.1083	223.1077	0.08	120.081; 103.055; 107.050; 91.055; 79.055	HMDB0028995
308	5.03	Ala-Phe	$C_{12}H_{16}N_2O_3$	237.1239	237.1233	-0.15	120.081; 121.084; 103.055; 107.049; 93.070; 91.054; 79.055	HMDB0028694
309	5.09	Trp	$C_{11}H_{12}N_2O_2$	205.0977	205.0973	0.35	118.065; 143.073; 132.081; 115.055; 144.081; 142.065	MSBNK-Washington_State_Univ-BML01197
310	5.30	Val-Leu	$C_{11}H_{22}N_2O_3$	231.1709	231.1703	-0.18	72.081; 55.055; 57.058	HMDB0029131
311	5.33	Thiamine (vit. B1)	$C_{12}H_{16}N_4OS$	265.1123	265.1117	-0.3	122.071; 81.045	HMDB0000235
312	5.68	γ-Glu-Leu	$C_{11}H_{20}N_2O_5$	261.1451	261.1445	-0.02	84.045; 86.097; 142.050; 130.050; 114.055	HMDB0011171
313	6.58	Trp-Val	$C_{16}H_{21}N_3O_3$	304.1661	304.1655	-0.48	72.081; 146.060; 55.055; 159.092; 144.081	HMDB0029096
314	7.19	Leu-Phe	$C_{15}H_{22}N_2O_3$	279.1709	279.1703	-0.66	86.097; 69.071; 120.081; 70.074; 57.058; 166.087	HMDB0013243
315	7.43	Leu-Trp	$C_{17}H_{23}N_3O_3$	318.1818	318.1812	-0.13	86.097; 69.071; 87.100; 159.092; 144.080	HMDB0028940
316	9.35	Indolo-3-carboxyaldehyde	C_9H_7NO	146.0606	146.0601	0.40	91.055; 117.057; 118.0658; 65.039; 90.047; 89.039	HMDB0029737

Ala, alanine; Arg, arginine; Asn, asparagine; Gln, glutamine; Glu, glutamic acid; Gly, glycine; His, histidine; Hpro, hydroxyproline; Ile, isoleucine; Leu, leucine; Lys, lysine; Met, methionine; Phe, phenylalanine; Pro, proline; Ser, serine; Thr, threonine; Trp, tryptophan; Val, Valine.
